# Variation in the ribosome interacting loop of the Sec61α from *Giardia lamblia*

**DOI:** 10.1186/s13062-015-0087-0

**Published:** 2015-09-30

**Authors:** Abhishek Sinha, Atrayee Ray, Sandipan Ganguly, Shubhra Ghosh Dastidar, Srimonti Sarkar

**Affiliations:** Department of Biochemistry, Bose Institute, P-1/12 CIT Road, Scheme VII M, Kolkata, 700054, West Bengal India; Molecular Parasitology, National Institute of Cholera and Enteric Diseases, P-33, C.I.T Road, Scheme XM, Kolkata, 700010, West Bengal India; Bioinformatics Center, Bose Institute, P-1/12 CIT Scheme VII M, Kolkata, 700054, West Bengal India

**Keywords:** *Giardia*, Sec61, Homology modeling, Ribosome, Endoplasmic reticulum, Molecular dynamics

## Abstract

The interaction between the ribosome and the endoplasmic reticulum-located Sec61 protein translocon is mediated through an arginine residue of Sec61α, which is conserved in all prokaryotic and eukaryotic orthologues characterized to date. Using *in silico* approaches we report that instead of arginine, this ribosome-interaction function is most likely discharged by a lysine residue in the protist *Giardia lamblia*. This functional substitution of the R with a K in GlSec61α may have taken place to accommodate a G-rich rRNA.

**Electronic supplementary material:**

The online version of this article (doi:10.1186/s13062-015-0087-0) contains supplementary material, which is available to authorized users.

## Findings

In eukaryotes, the heterotrimeric Sec61 complex (composed of α, β and γ subunits) forms a protein translocating channel across the endoplasmic reticulum (ER) membrane; co-translational protein import into the ER proceeds through this channel. The protein conducting channel is formed by the essential subunit Sec61α, which is composed of ten transmembrane helices. This ubiquitous protein is evolutionarily conserved and has undergone minimal structural change, even in comparison to its prokaryotic orthologue, SecY [1, 2].

Structural studies show that the cytoplasmic loop located between transmembrane helices 8 and 9 (loop 8/9) of Sec61α contains a conserved R residue that is present in all orthologues of Sec61α described thus far [3–5]. Cryo-EM studies of ribosome-bound Sec61α revealed that this R may interact with the helix H6 of the 5.8S rRNA and helix H53 of 28S rRNA [3, 4]. Here we report that the function of this R is likely to be discharged by a K residue (K426) in the Sec61α of the protist *Giardia lamblia* (GlSec61α). Sequence analysis, molecular modeling and simulation studies suggest that the molecular mechanism of ribosomal docking of GlSec61α is likely to be slightly altered compared to that in previously-characterized eukaryotes.  This is because the functional substitution of the R with a K in GlSec61α may have taken place to accommodate a change in sequence of the rRNA region that interfaces with loop 8/9. This indicates a possible coevolution of Sec61α and the ribosome.

## Predicted secondary structure of GlSec61α

**Fig. 1:**
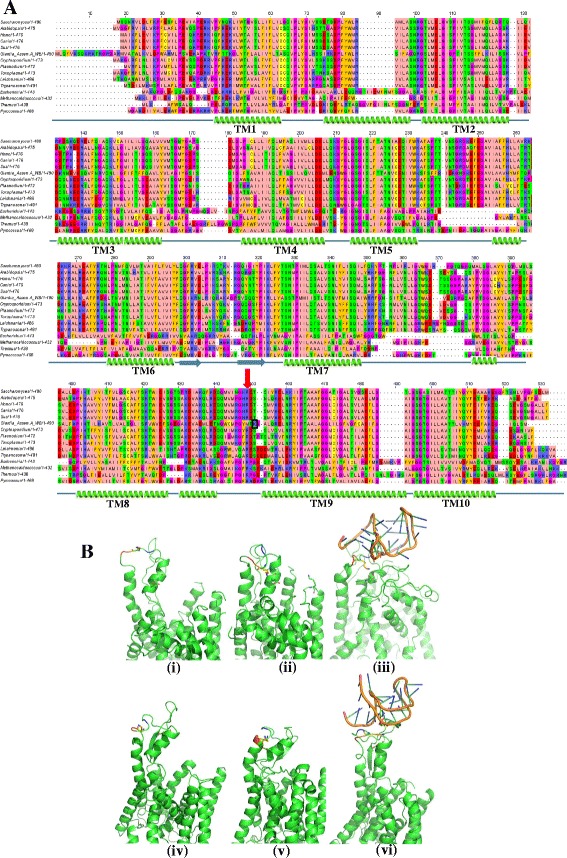
**a** Sequence alignment of GlSec61α from *G. lamblia* Assemblage A isolate WB with orthologous sequences from *S. cerevisiae, A. thaliana, H. sapiens, C. lupus, S. scrofa, C. hominis, P. falciparum, T. gondii, L. major, T. brucei, E. coli, M. jannaschii, T. thermophilus* and *P. furiosus*. The secondary structure elements have been marked below the alignment, with spirals representing α-helices, arrows representing β-strands and lines representing intervening loops. Only the transmembrane helices have been numbered. The downward pointing red arrow marks the conserved arginine (R) required for interaction with ribosome while the functionally-equivalent lysine (K) residue in the putative GlSec61α has been highlighted with a black box. **b** Tertiary structure of a section of GlSec61α obtained by homology modeling based on 2WWB (*i*, *ii* and *iii*) and 3J7Q (*iv*, *v* and *vi*). Each of the homology modeled structures underwent molecular dynamic simulation for 30 ns, with (*iii* and *vi*) or without (*ii* and *v*) docked RNA. The side chains of residues K426 and E414 are shown. To indicate the orientation of the loop 8/9, two residues on either side of K426 have been marked (424-*dark blue*, 425-*light blue*, 427-*amber* and 428-*red*).

Although the sequences of Sec61α orthologues are extremely conserved, GlSec61α has low sequence identity (between 34.7 % and 55.5 %) with the orthologous sequences derived from evolutionarily diverse eukaryotes (Additional file 1). To ensure that this divergent sequence indeed represents the Sec61α orthologue, we determined its predicted secondary structure and observed that similar to all eukaryotic Sec61α and prokaryotic SecY, GlSec61α has the potential to form ten transmembrane helices (Fig. 1a) [3–6]. The sequence alignment shows that the span of each helix and also the spacing between adjoining helices of GlSec61α are similar to that of other orthologues. Additionally, both Phyre2 and PSIPRED predict the N-terminus of the GlSec61α to be in the cytoplasm, which is identical to the topology of the other orthologues. Therefore, although the sequence of GlSec61α is least conserved amongst all the orthologues considered in this study, secondary structure predictions indicate that it is likely to adopt a similar structure.

## GlSec61α harbours functional substitution of a conserved arginine residue

The ribosome-interacting R residue in loop 8/9 is present in all prokaryotic and eukaryotic orthologues studied till date, including those from other protists (Fig. 1a) [7, 8]. However, sequence alignment shows that in GlSec61α, K426 is the only positively charged residue in the loop 8/9; thus it is most likely functionally equivalent to the R (Fig. 1a). This K is conserved in the GlSec61α orthologues from all the sequenced *Giardia* genomes (Assemblage E isolate P15, Assemblage A isolate WB, Assemblage B isolate GS_B, Assemblage A2 isolate DH and Assemblage B isolate GS) (Additional file 2). Interestingly, both K and R residues are present next to each other in the two putative Sec61α orthologues from another diplomonad, *Spironulceus salmonicida*, which is closely related to *Giardia* (Additional file 2).

As further support for K426 being involved in ribosomal interaction, we hypothesized that it will undergo conformational switching in a ribosome-dependent manner. Thus, we carried out molecular dynamic simulations on two sets of homology models (Model 1 with 2WWB.pdb and Model 2 with 3J7Q.pdb as independent templates, using implicit membrane environment), in presence or absence of a small fragment of rRNA docked as existing in 2WWB [3, 4]. In both cases since the template PDB originally had segments of rRNA attached to it, the homology modeling yielded a ‘target’ conformation of GlSec61α resembling the ribosome-complexed state (Fig. 1b, Panels *i* and *iv*). Each model underwent molecular dynamic simulation for 30 ns. The structures obtained at the end of the simulation indicate that in absence of the rRNA the charge of the K residue is likely to be stabilized through interactions with oppositely charged residues nearby (Fig. 1b, Panels *ii* and *v*). Thus, in both systems, K426 is mainly neutralized by E414. However, several other negatively charged residues, which are located further away, also participate in this interaction network in a many-body fashion. The approaching negatively charged rRNA, with its high density of phosphate groups, most likely causes a perturbation of this electrostatic interaction network. This notion finds support in a previous report documenting the stronger electrostatic influence of phosphate groups compared to the side chain of E residues [9]. Simulation of the RNA-docked structure indicated that loop 8/9 underwent ribosome-dependent movement whereby it was projected towards the ribosome but was more retracted when the ribosome was absent (Fig. 1b, compare panels *ii* with *iii* and *v* with *vi*). This substantial change in conformation of loop 8/9 caused a significant alteration in the position of the K residue and the simulations, based on both models, indicate that K426 switches towards the incoming RNA (Fig. 1b and Additional file 3). Thus, the K present in loop 8/9 undergoes conformational switching in a ribosome-dependent manner; therefore, it is likely to mediate the interaction between GlSec61α and the ribosome.

## Interaction between GlSec61α and ribosome involves non-canonical residues

Reported cryo-EM structures of mammalian Sec61α indicate that the conserved R in loop 8/9 forms specific interactions with the C2526 in H53 of 28S rRNA and the AGCG sequence present in the H6 stem-loop of 5.8S rRNA, which are both located at/near the universal adaptor site at the ribosomal tunnel exit [3, 4]. It is possible that given the divergent sequence of the rRNA of *Giardia* [10, 11], a change in an evolutionarily conserved residue of GlSec61α resulted from a necessity to interact with an altered ribosomal sequence. Perusal of the rRNA secondary structure (www.rna.icmb.utexas.edu) indicates that while the stem-loop structures of both the H53 and H6 are conserved in *G. lamblia* (5 bp stem with 8 nucleotide loop for H53 and 4 bp stem with 6 nucleotide loop for H6), the positions corresponding to both C2526 in H53 and the A in H6 are occupied by G in *G. lamblia* (both residues have been highlighted with yellow in Additional file 4). Alignment of the rRNA sequences of the eukaryotic species used in Fig. 1a indicates that in a majority of the sequences, the position corresponding to C2526 in H53 is occupied by either A or C, while all the 5.8S rRNAs, except *G. lamblia*, have an A in the H6 stem-loop (Additional file 4). The presence of G at both these positions of *G. lamblia* rRNA may be a consequence of the G-rich genome of this organism [12, 13]. Thus, it is possible that the necessity to optimize the interaction with a G residue in the ribosome may have resulted in the functional substitution of an R residue with K in *Giardia*.

## Possible subtle selectivity of K over R for interacting with G

The differences in physicochemical properties of amino acids and nucleotide bases may contribute towards this selectivity of K residue in proteins for interacting with G in RNA. The side chain of K is known to be different from that of R, both in terms of length and the nature of the functional group. The physicochemical characteristics of individual bases also differ. For example, although A and G are similar in size, they differ markedly in the number of H-bonds and van der Waals contacts formed, and also in their dipole moments; such differences are known to contribute significantly in the discrimination between A and G by nucleotide-binding proteins [14]. In addition, statistical data of RNA-protein interactions present in diverse organisms also indicate that while most RNA-protein interactions are mediated by R (which prefers A, C and U over G), if K is present, G is preferred because of increased number of van der Waals contacts [15]. This selectivity is further supported by a report documenting the coevolution of rRNA and ribosomal protein L22 showing that the substitution of a conserved R by K was accompanied by a change from U to G in the rRNA [16]. Thus, existing literature supports the notion that the functional substitution of R by K in GlSec61α may be a consequence of having to interact with the G-rich rRNA. However, this selectivity is likely to be so subtle that thermodynamic measurements may be unable to discern it and may only be significant when considered on an evolutionary time scale as it may create selection pressures. This may explain the observation that even though the R residue is evolutionarily conserved, yeast having R → K mutation in Sec61α exhibit no growth defect as ribosomal docking is possible even with a K residue [7].

## Conclusion

In conclusion, our analyses indicate that the interaction between GlSec61α and the ribosome is mediated by a K and not an R residue, which was hitherto thought to be invariant [3]. There is also an indication of RNA-protein coevolution as this replacement of R by K may be a compensatory change to accommodate a G-rich rRNA.

## Methods

### Sequence alignment of Sec61α subunit of *G. lamblia*

The Sec61α subunit of *G. lamblia* Assemblage A_WB was aligned with the orthologous subunits of *Saccharomyces cerevisiae, Arabidopsis thaliana, Homo sapiens, Canis lupus, Sus scrofa, Cryptosporidium hominis, Plasmodium falciparum, Toxoplasma gondii, Leishmania major, Trypanosoma brucei, Escherichia coli, Methanocaldococcus jannaschii, Thermus thermophilus,* and *Pyrococcus furiosus* using MUSCLE, MAFFT, ProbCons, KAlign and FSA [17–21] and a consensus alignment was built using META-COFFEE [22]; badly-aligned regions were manually masked using Jalview [23]. The transmembrane helix prediction was carried out using Phyre2 and PSIPRED [24, 25]. The pairwise sequence alignment for determining identity and similarity values of Sec61α from different eukaryotes was carried out using Pairwise Sequence Alignment tool of EBI (www.ebi.ac.uk/Tools/psa). The NCBI accession numbers of all the sequences used for the above-mentioned studies are provided in Additional file 5.

### Computational modeling of the structure of GlSec61α

The computational model of GlSec61α was constructed using SWISS-MODEL workspace [26]. The homology modeling was carried out separately with PDB structures having accession codes 2WWB and 3J7Q that have resolutions of 6.5 and 3.4 Å, respectively [3, 4]. A small fragment of rRNA that was adjacent to loop 8/9 in 2WWB was analogously docked onto each of the two structures of the *Gl*Sec61α homology models. The models without the rRNA were also prepared. Thus four systems were obtained. For simulations the systems were initially processed in the CHARMM-GUI web server [27]. All the systems were simulated using the CHARMM [28] simulation program, applying the CHARMM27 force field [29]. The GBSW model [30] of implicit water and membrane were used to represent the surrounding environment of the proteins. The implicit membrane had a 30 Å hydrophobic core slab and a 5 Å slab on either side to gradually switch the dielectric of the medium from the membrane to water. The homology modeled protein was inserted into the membrane aligning the helices approximately perpendicular to the membrane surface. Each structure was energy minimized (using ABNR method) and then was set for 30 ns simulations at 300 K, using the Langevin dynamics (LD) algorithm [31]. LD used a random force (set by FBETA 5.0 in CHARMM for all heavy atoms) to correspond to the frictions implied; it also ensured the collisions with a heat-bath kept at 300 K, to ensure a constant temperature of the system. The vibration of the bonds involving hydrogen atoms was frozen using SHAKE [32] which enabled the use of a 2 fs integration time step. The non-bonded interactions were smoothly switched to zero between 14 Å and 18 Å. Figures were prepared using Pymol [33].

### Reviewers’ comment

#### Reviewer 1: Dr. Srikrishna Subramanian

Sinha et. al., propose a homology model for the Giardia lamblia Sec61alpha protein using as template the 6.5 A cryo-EM structure of the canine Sec61alpha. Based on comparison of several eukaryotic homologs, they suggest that a highly conserved arginine proposed to interact with the 5.8S ribosome is mutated to a lysine. They suggest that this mutation is correlated to a corresponding A to G substitution in the 5.8 s rRNA sequence. They further argue that Giardia likely had two orthologues of Sec61alpha and lost one of them. Unfortunately the manuscript does not provide a deep and insightful analysis of the data and the evidence supporting their claims is not very compelling or convincing.

There are several major shortcomings:

1) Only a limited number of eukaryotic sequences are used in the analysis. My suggestion is to use prokaryotic sequences also in the analysis and study those in which the conserved arginine is mutated. The authors should also see how statistically correlated this change is with that of the interacting RNA residue.

Authors’ Response: We thank the reviewer for his suggestion regarding including prokaryotic sequences in the sequence analysis. In the revised manuscript we have included four prokaryotic sequences from *E. coli*, *T. thermophilus*, *M. jannaschii* and *P. furiosus*. While the first two are eubacteria, the last two belong to archaea. We have chosen these because the structures of their SecY are available. Sequence analysis shows that R is present in the loop 8/9 of these SecY proteins as well. Thus, even after the inclusion of prokaryotic sequences, it is evident that with the exception of *G. lamblia*, the arginine is present in loop 8/9. Sec61α of *D. rerio, D. melanogaster, C. elegans* and *P. ochrochloron*, although not included in this study, have been used in published sequence alignments [7, 8]; if these are taken into account then the number of organisms increases to 20, of which only one (*G. lamblia*) has K instead of R. Given the high levels of identity of metazoan sequences, we have also checked non-metazoans for which rRNA secondary structure is available (*Cryptococcus neoformans*, *Chlorella variabilis*, *Aedes aegypti*) [12] and observed that even in these cases R is present in loop 8/9 and also G does not occupy either of the two positions in the rRNA under consideration (marked in Additional file 4); given the limitation of space, these sequences could not be included in Fig. 1. To investigate statistically correlation between change in the residue in loop 8/9 and the interacting RNA residue, based on sequence data available for both Sec61α and rRNA, out of 11 organisms (Additional file 4), the null hypothesis ‘if R is present, then interacting residue can be G’ holds true for only 2 of 11 organisms in case of H53 and is untrue for all 11 organisms in case of H6.

2) The predicted secondary structural elements appear to be much shorter than those seen in the structures of homologs thus making the loops appear longer than they actually are in Fig. 1.

Authors’ Response: Although Sec61α is a transmembrane protein, it also has helices that do not span the membrane. We had only marked transmembrane helices in the figures. After the reading the reviewer’s comment, we realized that this fact may be overlooked by readers and we thank the reviewer of pointing this out to us. In the revised figure, we have shown all secondary structure elements but numbered the transmembrane helices only.

3) The lysine that is proposed to interact with rRNA does not align with the conserved arginine but is instead two residues away on the loop. The actual mutation appears to be a R to M rather than R to K. At best one could speculate that the K may play a similar functional role. This point needs to be kept in mind when considering how such a substitution might have occurred. In the section on “Possible mechanism of substitution of the critically-conserved R residue” the authors make a rather far-fetched speculation that Giardia might have once harbored two orthologous Sec61alpha genes allowing for the functional substitution in one of them with a concomitant mutation in the rRNA. A simpler explanation may be that the R to M mutation was functionally substituted by the K present further down the loop. Another explanation may be that such a mutation is compensated by other interactions. Also, there is no real evidence to call this a critically-conserved R as mutation of this residue to K in yeasts are not lethal.

Authors’ Response: To address the concern of the reviewer regarding the position occupied by K in GlSec61α *vis-á-vis* the R in the other orthologues, in the revised manuscript we have termed this a ‘functional substitution’. However, it may be noted that while the positions occupied by the K of GlSec61α and the R in the other orthologues do not match, these residues are present in a loop and not in any rigid secondary structure element. Since loops are more flexible, the K might easily perform the same function as an R without occupying an identical position in the sequence. Also, there is some variability in the length of the loop as the orthologues from *Plasmodium*, *Leishmania* and *Trypanosoma* have a slightly longer loop 8/9 compared to that of higher eukaryotes. Thus, as pointed out by the reviewer, the charge of the residue is likely to be more important than its precise position in the loop. In this context, it may be pointed out that the K residue is the only positively charged residue in the loop 8/9 of GlSec61α.

After taking into account the reviewer’s concern regarding the proposed mechanism of substitution of the R by K, we have removed this section from the manuscript.

The R residue has been referred to as ‘universally conserved’ in existing literature [3]. To address the reviewer’s comment, we have used the term ‘evolutionarily conserved’ instead of ‘critically conserved’.

4) How important is the contact made by R/K with the rRNA. Is this the only contact or are there other contacts? What other proteins interact with the rRNA? A comparison of bacterial and yeast Sec61alpha structures suggest that there are other residues and proteins that interact with the RNA. Also, there is no mention of the structural differences of this region among various homologs. This has a bearing on the MD simulation studies. Further, sequence alignment of the template used for homology modeling (Canine Sec61alpha) is not shown. How conserved are the amino acids between loops 8 and 9 in these sequences?

Authors’ Response: According to Voorhees et al., the contact between Sec61α and the ribosomal RNA is primarily mediated by loop 6/7 and loop 8/9, which are termed by them as ‘evolutionarily conserved’ [3]. The same study points out that the specificity is provided by the conserved R residue of loop 8/9 as it forms specific hydrogen bonding interactions with the rRNA, while they state that ‘very few specific hydrogen-bonding interactions are observed for loop 6/7’. The same scenario holds in case of prokaryotes as an atomic-resolution model of the prokaryotic ribosome-SecY channel complex, obtained through MDFF, shows that the interaction involving loop 6/7 and 8/9 ‘contribute to the majority of interactions’ [5]. This study further states that ‘In contrast to loop 6/7, loop 8/9-RNA hydrogen bonds almost exclusively engage the RNA backbone’. Thus although there are other contacts between the Sec61 translocon and the rRNA, the contact made by this R/K with the rRNA is extremely important. As mentioned previously, this loop8/9 is extremely conserved [3], and there is very little structural difference among various homologues. The *C. lupus* (used in 2WWB) and the *S. scrofa* (used in 3J7Q) Sec61α sequences have been included in the sequence alignment in the revised manuscript.

5) The sentence “When the highly negatively charged backbone of rRNA polynucleotide chain….with charge of the RNA backbone” (Page 2 second paragraph) is very speculative. What will trigger intra-protein disruption of charge-charge interactions and replace with another charge-charge interaction involving RNA sequence?

Authors’ response: The simulations carried out in presence of RNA have revealed that the K426 is exposed towards the rRNA in contrast to its intra-protein salt-bridged situation in the simulations carried out in the absence of RNA. It is plausible that due to the induction of the incoming rRNA, the intra-protein electrostatic interaction network gets perturbed. Since RNAs contain a high density of phosphate groups, they have a highly negatively charged surface and this is likely to have a stronger influence on K426, resulting in its release form the cage of salt bridge with glutamic acid. Such a predominance of the phosphate groups over that of Glutamic acid has been previously documented [9].

6) The structures used for homology modeling and consequently the models from MD are of poor resolution to clearly establish interaction between amino acid and nucleotides. This needs to addressed and discussed. Structure of Sec61 complexed with 80S ribosome (PDBID: 2WWB) is a cryo-EM structure solved at 6.5 A. At this resolution, it is highly unlikely that rotamer of R or K will be correctly captured. Authors have carried out molecular dynamics at 4 ns in implicit membrane environment in the absence of rRNA and other interacting proteins. I have concerns on MD being carried out on a modeled protein that has been built on a template with poor resolution. Initial structure for the MD simulations is unlikely to be a local minimum structure. Details of energy minimization/MD parameters and setting up (heating, equilibration etc.) of system for MD run have not been provided. Time scale is too small to interpret changes in loop regions of protein arising due to mutations. Larger time scales (more than 30 ns) MD simulations on proteins with “R” as well as “K” at appropriate positions may establish flipping of K etc. in a properly setup system. Further, in the absence of RNA during MD, interaction between aminoacids and nucleotides cannot be discerned.

Authors’ response: As mentioned in the text, we have carried out molecular dynamic simulations on two sets of homology models using PDB structures 2WWB (6.5 Å) and 3J7Q (3.4 Å) as templates. Each model contained a small fragment of rRNA attached on the loop 8/9. The corresponding models without the rRNA were also prepared and each of the four models was run for 30 ns. Hence each system was started from two independent points of the conformational spaces and provided independent sampling totalling to 60 ns, which we believe to be satisfactory for the purpose mentioned by the reviewer. This has also provided the opportunity to test the effect of the difference in the resolution of the model-templates on the observed dynamics. Details of the methodology have been included in the Methods section.

7) In spite of being a short Discovery Report, the manuscript is difficult to read and crucial information such as residue numbers, details of methods to repeat the study are scattered around the manuscript or are completely missing.

Authors’ Response: We have tried our level best to improve the quality of English and also to address the lacunae that were pointed out by the reviewer.

Quality of written English: Not suitable for publication unless extensively edited.

#### Reviewer 2: Prof Piotr Zielenkiewicz

The manuscript describes the putative interaction pattern between ribosome and the Sec61# transmembrane protein from Giardia lamblia. As authors note, Giardia family has unique interacting loop mutation R- > K and at the same time another substitution on 5.8S rRNA sequence (A- > G). Authors conclude that both substitutions are due to coevolution and compensation of such modifications of both entities. The whole manuscript is very speculative and authors provide very little evidence to support their claims, which (one must admit) seem to be logical. I would like to raise some major issues which should be addressed before publication.

Major revisions:

# The proposed interaction change should be strongly supported by data included in the MS. Otherwise it is only a speculation. Molecular dynamics simulation which was carried out as a part of this work sheds little light, if any, on the proposed mechanism of interaction. It was surprising that the rRNA was entirely removed from this simulation, since it should be of the main interest. MD simulations with rRNA would be strongly recommended.

Authors’ Response: There are several studies that document that the R residue of loop 8/9 is crucial for the binding between the ribosome and Sec61α. As previously mentioned in our response to Reviewer 1’s comment, in contrast to the positively-charged residues of loop 6/7, this R residue of loop 8/9 is responsible for specificity. This R residue is present in all Sec61α and SecY orthologues published till date and this is evident even in the sequence alignments published in many of these studies [7, 8]. Thus this R residue has even been termed to be ‘universally conserved’ [3]. Inspection of the sequence of loop 8/9 of the *G. lamblia* orthologue shows that there is only one positively charged amino acid, which is K426. Its position in the loop is also comparable to that occupied by the R residue in the other orthologues. Thus based on similarity of charge and also the position, the K426 is most likely to be the residue that interacts with the ribosome. This has been pointed out in the manuscript.

As per the reviewer’s suggestion, we have carried out MD simulations in the presence of a fragment of rRNA that has been docked onto Sec61α. The data documenting the difference in the position of the K426 after 30 ns, in presence and absence of the RNA, provides support for the proposed interaction.

# Through entire manuscript the Authors state, that there is “change in amino acids R- > K”, but those residues are marked on sequence alignment to be in different places. It should be clearly noted that the change is not simply a mutated residue, but in fact two independent mutations within one loop.

Authors’ Response: We have already addressed this concern as it was also raised by Reviewer 1. Kindly see the response to question number 3.

# “Materials and methods” section needs be more precise and descriptive. Only brief and incomplete information is given to the reader about calculations carried out in the manuscript.

Our response: The details have now been included in the methods section.

Minor revisions:

# “Findings” section needs introduction paragraph briefly describing what is the “essential nature of its [Sec61] function”.

Authors’ Response: As per the reviewer’s suggestion, we have altered the introduction paragraph.

# Are A. thaliana and H. sapiens the only other eukaryotic (besides protists) orthologs of Sec61#? It should be noted why only those two are included in the paper.

Authors’ Response: In the interest of space, we had included only these two orthologues. However, the revised manuscript contains several more eukaryotic and also prokaryotic sequences. Additionally, we have also included references to previously-published sequence alignments that include other orthologues as well [7, 8].

# MD software is not mentioned by name, one can only guess that it was NAMD, wasn’t it? This is strange in contrast to both programs to generate pictures and movies which are cited.

Authors’ response: The details have been provided in the methods section. The software used was CHARMM.

# Why MD simulation stopped after 4 ns? What was the RMSD distribution after such time? Authors should comment on that.

Authors’ response: To address this concern the simulation has been extended to 30 ns for each trajectory, totalling to 120 ns. We believe that this amount of sampling is sufficient for the stated objective. The RMSD plots have been provided in the supporting information (Additional file 3).

# Currently there seem to be better structures to select as templates in the PDB, eg. 3j7q (09.2014). Authors could elaborate in few sentences why they’ve chosen their template (from 2009). Also, considering the level of identity, choice of homology modeling software is dubious, but for MD simulation it could be not that much relevant. Few words of explanation would be nice.

Authors’ response: As per the reviewer’s suggestion, a model based on 3J7Q has been included, in addition to the one based on 2WWB. We have based our conclusions on the results obtained from both structures. Even though the sequence identity between GlSec61α and the template models’ sequence is fairly low (Additional file 1), the modeled structures remain stable even over 30 ns; this stability corroborates the reliability of the resulting structures.

# Authors should use full organism names throughout manuscript where they meant specific organism (e.g. Additional file 1, Fig. 1a).

Authors’ Response: We have made these changes.

# Additional files:

# File 2: Movie generated from MD simulation should be of better quality.

Authors’ response: Originally we had carried out MD simulations using a single homology-modeled structure based on 2WWB.pdb. Since the revised manuscript contains MD simulations of two homology-modeled structures, we feel that the movie of the MD simulation is no longer necessary. Thus we have omitted this additional file.

# File 3: I believe that “side view”, should also be included (similar to the one in Additional file 2). Otherwise “hydrophobic parts” are not clearly visible.

Authors’ response: As previously mentioned above, since the revised manuscript contains MD simulation based on two independent homology models, we have removed the contents of the previous Additional file 3 as well.

Quality of written English: Acceptable.

## Reviewers comments after second round of review

### Reviewer 1: Dr. Srikrishna Subramanian

Only a limited number of sequences are used in the analysis. I had previously suggested that additional sequences, especially those in which the arginine is mutated to a lysine should be studied in order to validate the claim that this mutation is correlated with the change of A to G in the rRNA. The authors added four more sequences to their alignment and claim that Giardia is the only organism in which the arginine is functionally substituted by a lysine.

Our response: In light of the reviewer’s previously-expressed concern regarding limited number of sequences, we had added six new sequences (from *C. lupus*, *S. scrofa*, *E. coli*, *M. jannaschi*, *T. thermophilus* and *P. furiosus*) to the alignment in Fig. 1, which now had a total of 15 sequences. The number was low as we had restricted the new additions to only those proteins whose structure was known (as clearly stated in our previous response). Availability of structural information was crucial as we wanted to base our identification of loop 8/9 in the context of the position it occupies in the structure. There are several putative Sec61alpha orthologues in various sequence databases; however, many of these have been identified only on the basis of sequence homology alone and the corresponding genes may not be functional. In fact several hits that are obtained after BLAST searches with the *Giardia* protein are actually termed as ‘uncharacterized protein’ (7 of the 26 sequences in the reviewer’s dataset fall in this category). We did not want such sequences in our training dataset. Also, even though high-throughput data in the Giardia genome database documents change in the expression pattern of the corresponding gene, we ensured that this is not a pseudogene by carrying out RT-PCR analysis in our laboratory and also subjected the homology modeled structure to MD simulations to ensure that it is a functional orthologue. Furthermore, we had clearly mentioned that if previously published sequence alignments are taken into consideration then the total number of sequences goes up to 20. In fact this number is also mentioned by the reviewer himself at the beginning of his latest review. Thus our ‘claim’ was based on comparing the *Giardia* sequence to 19 other sequences which have been previously cited in literature. Also one needs to keep in mind the size limit of the manuscript.

With respect to claiming that *Giardia* is the only organism in which the R is functionally substituted by a K, it may be noted that the primary point of the manuscript is to draw the readers’ attention to the fact that the R residue of loop 8/9 is not invariant, which is contrary to the previously established idea (this is clearly stated in the conclusion section). Since considerable research efforts have already been directed towards understanding the structure and function of this protein, it is imperative to make the research community aware that the R residue is not invariant. The presence of other orthologues in which no R residue is present in loop 8/9 lends support to our argument. In fact the sequence logo for all the sequences obtained after six iterations of jackhmmr with GlSec61α sequence as query, shows that in less than 50 % cases, the position may also be occupied by K, Q or M. But again many of these sequences belong to uncharacterized proteins and it will not be worthwhile to determine whether an R is present in the close vicinity of this K/Q/M. Thus the overall conclusion remains that GlSec61α belongs to a group of a small number of sequences that do not contain any R in loop 8/9. In fact only 5 of the 26 sequences sent by the reviewer satisfy this condition. So the fact remains that sequences without R in loop 8/9 constitute a minority; however, for the purpose of this manuscript, we do not feel that there is any need to document all of them.

We would like to thank the reviewer for sending us an alignment of 26 sequences in which that particular position is not occupied by R. However, although it was mentioned in the review that these are eukaryotic orthologues, it may be noted that 8 of these are homologous to the prokaryotic SecY as these are encoded by the chloroplast genome (WP_009524363, A0A075C0M6, P28540, A0A075DWT6, B7T1W7, W0RZF8, J7F5U6 & A0A0B5W361) and another 7 are uncharacterized (A0A087XL37, A0A0D3CEH6, M5VMG0, D8TUF2, D8QPR1, W5AID7 & A5C0J8); also the sequence from *Oryza* (Q0J0G0) does not correspond to the Sec61alpha of this plant; the putative Sec61alpha of *Oryza* has a GenBank ID of AAT76995.1, and also contains the conserved R. Many of these sequences from Uniprot are actually found to be incomplete and in some when the entire sequence was retrieved from NCBI and aligned, R was found to be located in the loop 8/9 (the sequences and the MSA is provided for perusal in Additional file 6).

As I mentioned during my earlier review, the K that is proposed to interact with rRNA in *Giardia* does not align with the conserved R but is instead two residues away on the loop. The actual mutation appears to be an R- > M rather than R- > K. In fact as is now evident there are many sequences in which this R is substituted by a K or by other neutral or negatively charged residues. In fact one of these proteins where a true (as inferred from the sequence alignment) substitution of R to K is seen is that of *Spironucleus salmonicida* which the authors report in their paper (Additional file 2) but fail to identify it as an R- > K substitution due to the presence of a neighbouring R.

Our response: We had addressed this comment in our previous response where we stated that the K is termed as a functional substitution; we had also provided substantial clarifications regarding this matter. We would like to state once again that the presence of K, in conjunction with the absence of any R residue, in loop 8/9 is observed for only a small subset of Sec61alpha orthologues. It is not enough to just document the presence or absence of a given amino acid at this particular position; rather one has to also consider the distribution of charged residues over the entire loop 8/9. The reviewer may refer to his own alignment and observe that only 1 out of the 26 sequences, which themselves represent a minor population of Sec61alpha orthologues, does not contain any charged residues in loop 8/9 (B7T1W7). In case of *Spironucleus salmonicida*, it may be noted that an R is present right after the K (as also pointed out by the reviewer) and it will contribute substantially to the electrostatic field and also interact with the incoming RNA.

The possibility of the M playing the predominant role of recruiting and binding RNA, rather than the K located only two amino acids away, is not supported by a large body of existing literature that are based on multiple crystal structures of RNA-protein complexes. For example, it is known that histidine, arginine, threonine and lysine have the highest propensity to bind with RNA (Jeong et al. (2003) Mol. Cells, 16, 161–167), which reveals that polar interactions dominate the binding. Also, multiple studies, based on crystal structure of RNA-protein complexes, have documented that positively-charged residues are at the very least 10 times (both in terms of number and area) more likely to occupy RNA-protein interfaces compared to M (Bahadur et al. (2008) Nucleic Acids Research, 36, 2705–2716; Barik et al. (2015) J Biomol Struct Dyn. In press). The only way to conclusively determine which of the two residues (M or K) is more important for RNA binding is to take a genetic approach and assess the effect of each mutation on the viability of *Giardia*. However, the tetraploid nature of the *Giardia* genome makes such experiments impossible to conduct as it will never be possible to delete all four copies of the endogenous *glsec61α* gene.

The authors could obtain more such examples of genuine R- > K substitutions and test in each case if it is accompanied by a change of A to G in the 5.8S rRNA.

Our response: We carried out BLAST searches against eukaryotic genomes and identified the Sec61alpha orthologue of the microsporidia *Capsaspora owczarzaki* as having only K in loop 8/9 (this is also present in the reviewer’s data set). However, since the structure of the rRNA of this organism is not available, it is difficult to identify the base occupying the corresponding positions in the rRNA. Thus is the absence of reliable rRNA structure it is not possible to test this possibility.

Given that the R is not absolutely conserved, another speculation is that the K is not a functional substitute in the Giardia protein and this protein may not bind rRNA via this interface?

Our response: This is an interesting speculation and such criticism can be directed against all studies involving proteins of all those organisms that are recalcitrant to genetic manipulations or are uncultivable. Using such a yardstick will call into question the very approach of sequence analyses. But one also needs to keep in mind that given the fact that Sec61alpha has remained extremely conserved, both structurally and functionally, the likelihood that substantial functional alterations may have taken place whereby there has been a complete loss of function of loop 8/9 seems to be a remote possibility, especially because the loop’s sequence indicates that it may be able to discharge the function of ribosome binding.

I reiterate that the structures used for homology modeling and consequently the models from MD are of poor resolution to clearly establish interaction between amino acid and nucleotides. Thus the part dealing with MD is mostly unreliable and all that one can speculate is that the lysine possibly interacts with the RNA.

Our response: Following the reviewers’ suggestion, in our first round of revision we have already presented a 2nd set of calculations based on a PDB structure with resolution of 3.4 Å (3J7Q). In fact this model was suggested by the second reviewer, Prof. Zielenkiewicz. Of all the available templates in PDB presently, this structure has the best resolution (the best resolution of ribosome-SecY complex is only 7.1 Å). Furthermore, the use of a model of a resolution poorer than 3.4 Å is not uncommon in the field of molecular simulation. One primary justification is that MD itself acts as a tool for structure refinement. So within the limit of accuracy of the molecular mechanical force field, the structures get refined during the equilibration of the system. As the system experiences molecular dynamics it really doesn’t matter whether the initial resolution was 1.0 or 3.0 as all the atoms starts changing their positions once the MD is invoked.

The authors state that if K is present, G is preferred because of the increased number of van der Waals (minor note: Waals not Waal) contacts.

Are these increased contacts observed in the MD model? Where does the loop interact with the RNA?

Our response: To compare this we needed models of appropriate mutants, in complexed and uncomplexed states. This would be a rigorous exercise just to quantify the difference in van der Waals interactions that only act over short distances. Here the precision of docking determines the accuracy of such quantitative measurements. Therefore, only the experimentally determined, high-resolution structures of Sec61-RNA complex (and corresponding mutants) would have been appropriate for such quantitative estimations. Thus, in this case, how reliable would these extensive calculations be? The reviewer has already expressed his reservations regarding the resolution of the models. So to circumvent these concerns, we had based our conclusions on statistically interpreted experimental data available in literature, rather than attempting to extract the tiny differences in van der Waals energy from the MD data.

I find the following statements made in response to my previous comments contradictory:

In response to my third question:

However, it may be noted that while the positions occupied by the K of GlSec61α and the R in the other orthologues do not match, these residues are present in a loop and not in any rigid secondary structure element. Since loops are more flexible, the K might easily perform the same function as an R without occupying an identical position in the sequence.

In response to my fourth question:

As mentioned previously, this loop 8/9 is extremely conserved [3], and there is very little structural difference among various homologues.

Our response: The reviewer raised concerns regarding contradictions in our response, *viz.* the loop 8/9 is extremely conserved and there is very little structural difference among various homologues. We also stated that the loop 8/9 is flexible and the position of K in GlSec61alpha does not match with the conserved R of the other orthologues. In our statement we have used the term ‘conserved’ in context of the amino acids in that particular stretch forming a loop and not any other secondary structural element. ‘Conserved’ was not use either in the sense of the order of the amino acid sequences within the loop itself, or in terms of the length of the loop. From our alignment it is evident that *Plasmodium*, *Leishmania*, *Trypanosoma*, *Methanocaldococcus*, *Pyrococcus* and *Giardia* exhibit slightly longer loops than that present in higher eukaryotes. But in all of them, except *Giardia*, the R residue is present in the same position of the alignment. If the position of the R residue in the context of the length of the loop would have been vital then the R residue should have be positioned further down the loop 8/9 of all the above-mentioned orthologues. However, since this is not the case, it clearly indicates that the flexibility of the loop allows R to interact with the incoming ribosome.

This study further states that ‘In contrast to loop 6/7, loop 8/9-RNA hydrogen bonds almost exclusively engage the RNA backbone.

If the interaction is with the RNA backbone then why is the A to G change in the rRNA important?

Our response: The conclusion stated here was taken from a paper reporting the structure of SecY at 9.6 Å (PDB ID: 3KC4 and 3KCR). As mentioned in our previous response, a latter study by Voorhees et al. [3] with the Sec61alpha (3.4 Å) clearly established that the R residue of loop 8/9 forms specific interaction with a specific base of rRNA. We hope that this satisfactorily eliminates any confusion regarding this matter.

Quality of written English: Not suitable for publication unless extensively edited.

Our response: Once again, we have tried our level best to address this concern.

Reviewer 2 had no additional comments.

## Additional files

Additional file 1: Table S1. Identity percentage values between different Sec61α orthologues. (DOCX 74 kb) Additional file 2:
**Multiple sequence alignment of GlSec61α from** ***G. lamblia*** **Assemblage A isolate WB, Assemblage B isolate GS, Assemblage A2 isolate DH, Assemblage B isolate GS_B and Assemblage E isolate P15 with the two orthologous sequences from** ***S. salmonicida*** **.** Downward blue arrow marks the K residue in loop 8/9 of the GlSec61α orthologues, while the upward red arrow marks the R residue in the same loop of Sec61α orthologues from *S. salmonicida*. (PPTX 197 kb) Additional file 3:
**Tertiary structure of GlSec61α obtained by homology modeling based on 2WWB (a & b) and 3J7Q (c & d), followed by molecular dynamic simulation for 30 ns, with (b & d) or without (a & c) docked RNA.** Other details are same as those described in Fig. 1b. The corresponding RMSD graphs are shown below each structure. The templates used for modeling (2WWB and 3J7Q) had several other protein/peptide chains and RNA fragments, all of which exerted a constraint on the protein conformation. Thus, although the homology modeled structures resembled that conformation of the template, the above-mentioned constraints were absent in our simulations as only a fragment of the RNA was used for docking. As a result, during simulation, the structures relaxed in the first few nanoseconds and this resulted in a rise of the RMSD before subsequent stabilization. Hence observed increase of RMSD does not reflect any destabilization. (PPTX 1691 kb) Additional file 4: Figure S1. (A) Helices H51 and H53 of the 28S ribosomal RNA of *Homo sapiens* (left panel) and *G. lamblia* (right panel). (B) Helices H5, H6 & H7 of 5.8S rRNA of large ribosomal subunit of *Homo sapiens* (left panel) and *G. lamblia* (right panel). Sequences highlighted in yellow in (A) and (B) represent residues of H53 and H6 that interact with the R present in loop 8/9 of Sec61α. The ribosomal structure is derived from Comparative RNA Website and Project (www.rna.icmb.utexas.edu). Sequence alignments of the corresponding regions of the 28S rRNA and 5.8S rRNA, derived from *Saccharomyces cerevisiae, Arabidopsis thaliana, Homo sapiens, Canis lupus, Sus scrofa, Giardia lamblia, Cryptosporidium hominis, Plasmodium falciparum, Toxoplasma gondii, Leishmania major* and *Trypanosoma brucei* are shown below. The residues highlighted in yellow in the RNA secondary structure have been marked with a black box in the alignments. Colour scheme was according to ClustalX. (PPTX 93 kb) Additional file 5: Table S2. NCBI accession numbers of Sec61α sequences used in this study. (DOCX 30 kb) Additional file 6:
**MSA of Sec61α sequences.** (PDF 188 kb)

## Abbreviations

ER: Endoplasmic reticulum
Cryo-EM: Cryo electron microscopy
PDB: Protein data bank
LD: Langevin dynamics
