# Supplementary material for: Variation in the ribosome interacting loop of the Sec61α from Giardia lamblia
Source: Biol Direct. 2015 Sep 30;10:56. doi: 10.1186/s13062-015-0087-0 (PMC4588681; doi:10.1186/s13062-015-0087-0)
Supplement: Additional file 1: Table S1. — Identity percentage values between different Sec61α orthologues. (DOCX 74 kb) [file 13062_2015_87_MOESM1_ESM.docx]

Table S1: Percent identity scores between Sec61α subunits of different eukaryotes

| ***Saccharomyces***  ***cerevisiae*** | ***Arabidopsis***  ***thaliana*** | ***Homo***  ***sapiens*** | ***Canis***  ***lupus*** | ***Sus***  ***scrofa*** | ***Giardia***  ***lamblia*** | ***Spironucleus***  ***salmonicida*** | ***Cryptosporidium***  ***hominis*** | ***Plasmodium***  ***falciparum*** | ***Toxoplasma***  ***gondii*** | ***Leishmania***  ***major*** | ***Trypanosoma***  ***brucei*** |
| --- | --- | --- | --- | --- | --- | --- | --- | --- | --- | --- | --- |
| ***Saccharomyces***  ***cerevisiae*** | **54.7** | **56.8** | **54.7** | **54.9** | **37.4** | **35.8** | **53.8** | **52.7** | **53.8** | **46.3** | **45.4** |
|  | ***Arabidopsis***  ***thaliana*** | **66.7** | **66.5** | **66.7** | **40.6** | **39.8** | **68** | **65.9** | **68** | **52.8** | **54** |
|  |  | ***Homo***  ***sapiens*** | **99.8** | **100** | **38.4** | **38.9** | **58.7** | **66.5** | **68.7** | **55.6** | **53.1** |
|  |  |  | ***Canis***  ***lupus*** | **99.8** | **38.4** | **37.9** | **66.6** | **65.3** | **67** | **55.4** | **52.9** |
|  |  |  |  | ***Sus***  ***scrofa*** | **38.4** | **37.9** | **66.8** | **65.6** | **67.2** | **55.6** | **53.1** |
|  |  |  |  |  | ***Giardia***  ***lamblia*** | **55.5** | **39.6** | **42.1** | **40.7** | **37** | **34.7** |
|  |  |  |  |  |  | ***Spironucleus***  ***salmonicida*** | **37.4** | **38.4** | **37.9** | **36.5** | **36** |
|  |  |  |  |  |  |  | ***Cryptosporidium***  ***hominis*** | **78.1** | **83.7** | **56.6** | **53.7** |
| The values corresponding to *G. lamblia* have been underlined |  |  |  |  |  |  |  | ***Plasmodium***  ***falciparum*** | **80** | **54.7** | **53.7** |
|  |  |  |  |  |  |  |  |  | ***Toxoplasma***  ***gondii*** | **56.2** | **54.4** |
|  |  |  |  |  |  |  |  |  |  | ***Leishmania***  ***major*** | **77** |
|  |  |  |  |  |  |  |  |  |  |  | ***Trypanosoma***  ***brucei*** |
