# Supplementary figures and images for: Variation in the ribosome interacting loop of the Sec61α from Giardia lamblia
Source: Biol Direct. 2015 Sep 30;10:56. doi: 10.1186/s13062-015-0087-0 (PMC4588681; doi:10.1186/s13062-015-0087-0)

## Slide 1
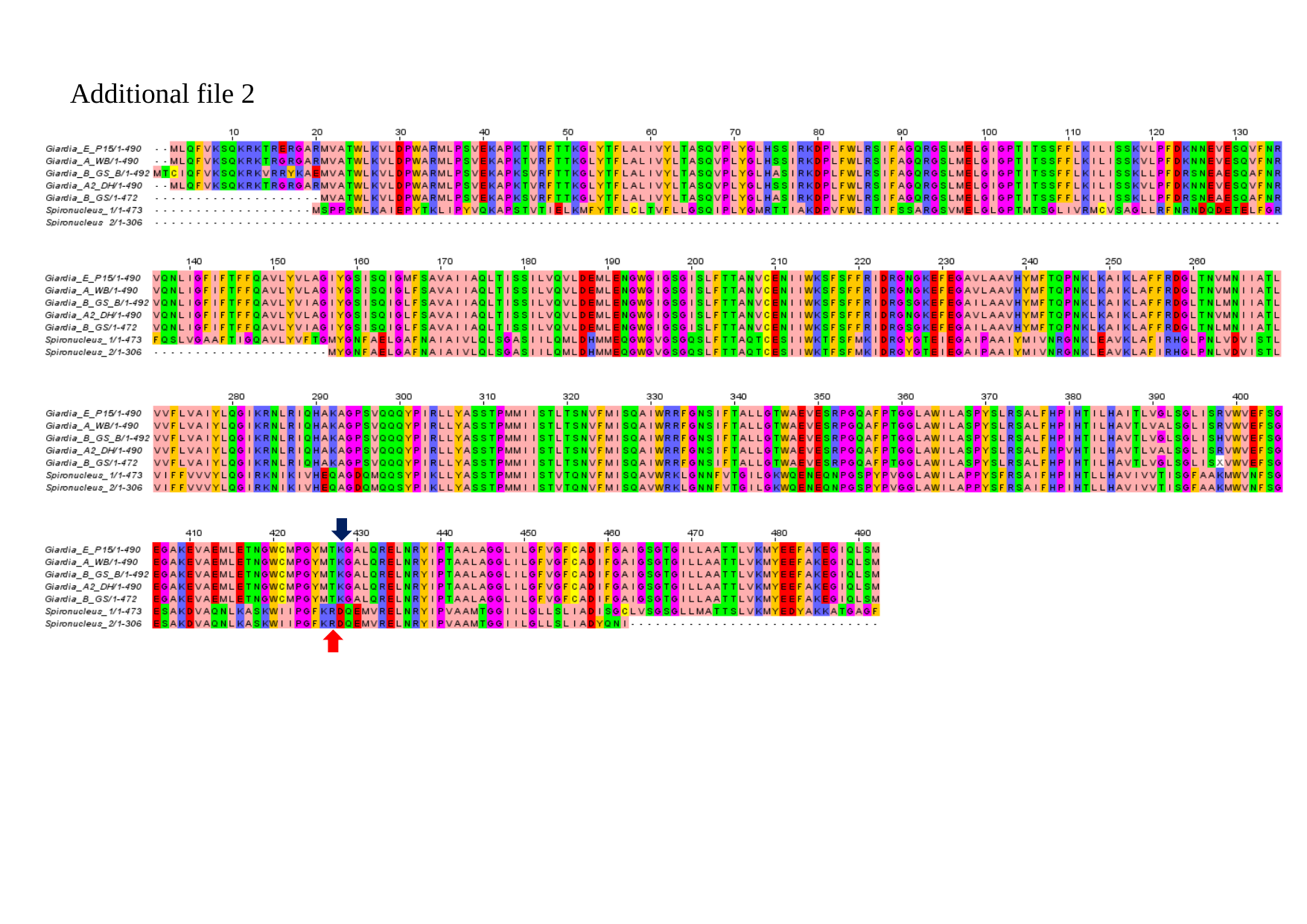

Additional file 2

Supplement: Additional file 2: — Multiple sequence alignment of GlSec61α from G. lamblia Assemblage A isolate WB, Assemblage B isolate GS, Assemblage A2 isolate DH, Assemblage B isolate GS_B and Assemblage E isolate P15 with the two orthologous sequences from S. salmonicida . Downward blue arrow marks the K residue in loop 8/9 of the GlSec61α orthologues, while the upward red arrow marks the R residue in the same loop of Sec61α orthologues from S. salmonicida. (PPTX 197 kb) [file 13062_2015_87_MOESM2_ESM.pptx]

## Slide 1
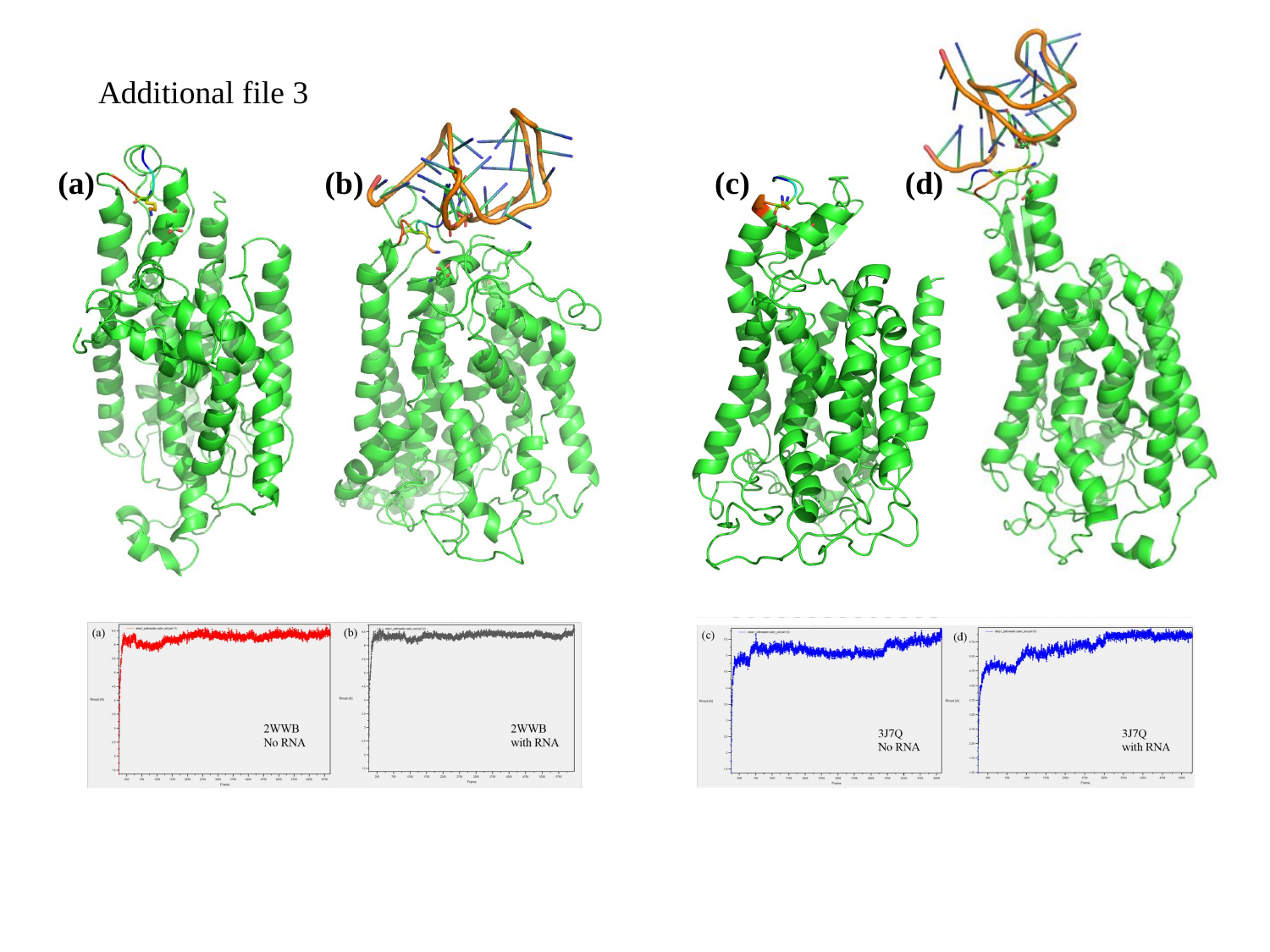

Additional file 3
(a)
(b)
(c)
(d)

Supplement: Additional file 3: — Tertiary structure of GlSec61α obtained by homology modeling based on 2WWB (a & b) and 3J7Q (c & d), followed by molecular dynamic simulation for 30 ns, with (b & d) or without (a & c) docked RNA. Other details are same as those described in Fig. 1b. The corresponding RMSD graphs are shown below each structure. The templates used for modeling (2WWB and 3J7Q) had several other protein/peptide chains and RNA fragments, all of which exerted a constraint on the protein conformation. Thus, although the homology modeled structures resembled that conformation of the template, the above-mentioned constraints were absent in our simulations as only a fragment of the RNA was used for docking. As a result, during simulation, the structures relaxed in the first few nanoseconds and this resulted in a rise of the RMSD before subsequent stabilization. Hence observed increase of RMSD does not reflect any destabilization. (PPTX 1691 kb) [file 13062_2015_87_MOESM3_ESM.pptx]

## Slide 1
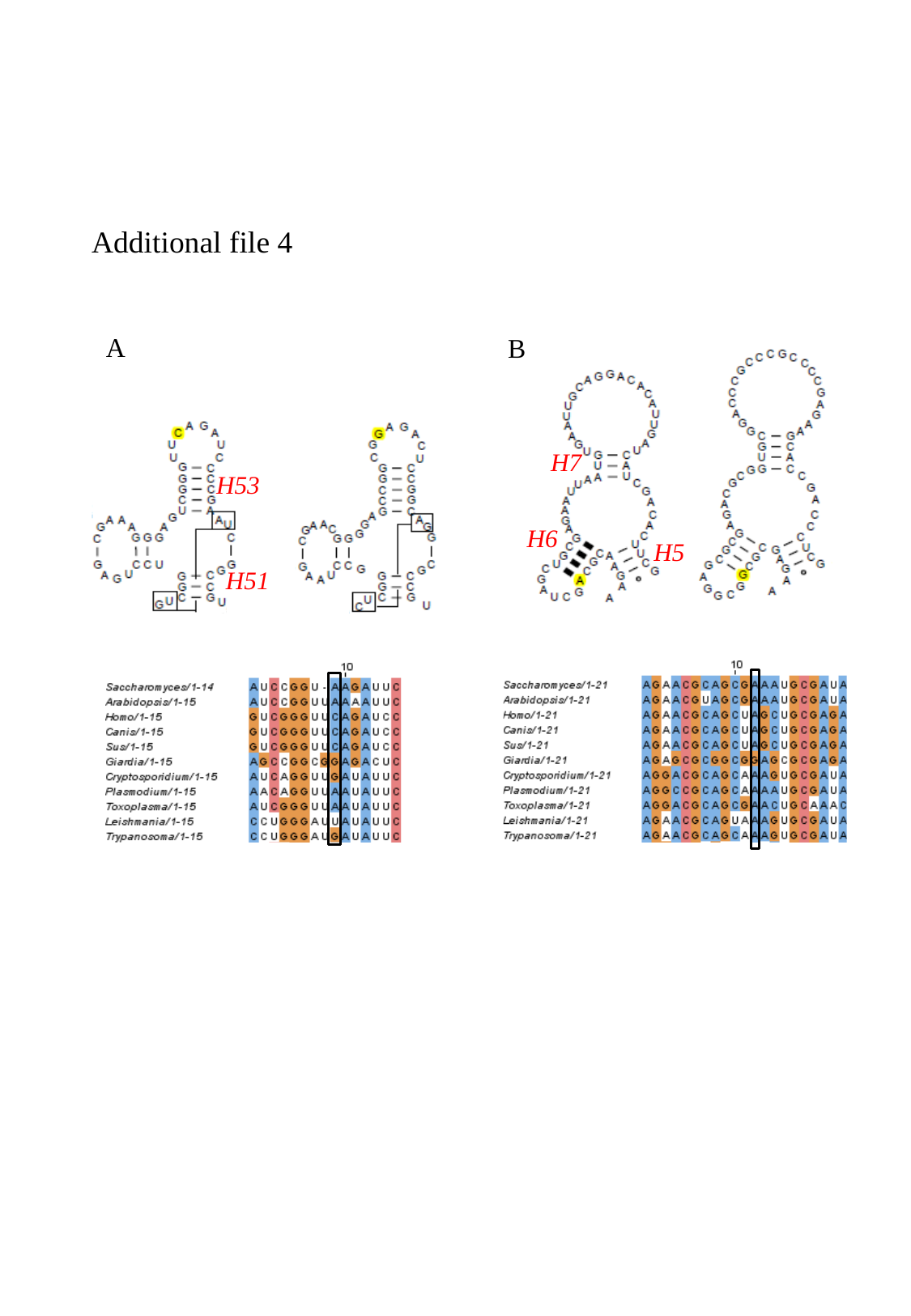

Additional file 4
A
B
H7
H6
H5
H53
H51

Supplement: Additional file 4: Figure S1. — (A) Helices H51 and H53 of the 28S ribosomal RNA of Homo sapiens (left panel) and G. lamblia (right panel). (B) Helices H5, H6 & H7 of 5.8S rRNA of large ribosomal subunit of Homo sapiens (left panel) and G. lamblia (right panel). Sequences highlighted in yellow in (A) and (B) represent residues of H53 and H6 that interact with the R present in loop 8/9 of Sec61α. The ribosomal structure is derived from Comparative RNA Website and Project (www.rna.icmb.utexas.edu). Sequence alignments of the corresponding regions of the 28S rRNA and 5.8S rRNA, derived from Saccharomyces cerevisiae, Arabidopsis thaliana, Homo sapiens, Canis lupus, Sus scrofa, Giardia lamblia, Cryptosporidium hominis, Plasmodium falciparum, Toxoplasma gondii, Leishmania major and Trypanosoma brucei are shown below. The residues highlighted in yellow in the RNA secondary structure have been marked with a black box in the alignments. Colour scheme was according to ClustalX. (PPTX 93 kb) [file 13062_2015_87_MOESM4_ESM.pptx]
