# Supplementary material for: Variation in the ribosome interacting loop of the Sec61α from Giardia lamblia
Source: Biol Direct. 2015 Sep 30;10:56. doi: 10.1186/s13062-015-0087-0 (PMC4588681; doi:10.1186/s13062-015-0087-0)
Supplement: Additional file 5: Table S2. — NCBI accession numbers of Sec61α sequences used in this study. (DOCX 30 kb) [file 13062_2015_87_MOESM5_ESM.docx]

Table S2: NCBI accession number of Sec61α (eukaryotes) and SecY (prokaryotes) sequences used in this study

| **Organism name** | **NCBI accession no.** |
| --- | --- |
| *Saccharomyces cerevisiae* | [GenPept:AAB67276] |
| *Arabidopsis thaliana* | [GenPept:NP_177993.1] |
| *Homo sapiens* | [GenPept:NP_037468.1] |
| *Canis lupus* | [GePept:NP_001003315.1] |
| *Sus scrofa* | [GenPept:NP_001231283] |
| *Giardia lamblia* Assemblage A isolate WB | [GenPept:XP_001705341.1] |
| *Spironucleus salmonicida* | [GenPept:EST42237] |
| *Cryptosporidium hominis* | [GenPept:XP_665568.1] |
| *Plasmodium falciparum* | [GenPept:CAD52584] |
| *Toxoplasma gondii* | [GenPept:XP_002365676.1] |
| *Leishmania major* | [GenPept:CAJ02934.1] |
| *Trypanosoma brucei* | [GenPept:EAN79585.1] |
| *Escherichia coli* | [GenBank: EGT69311.1] |
| *Methanocaldococcus jannaschii* | [Swiss-Prot: Q60175.2] |
| *Thermus thermophilus* | [GenBank: BAC01134.1] |
| *Pyrococcus furiosus* | [GenBank: AAL81925.1] |
| *Giardia lamblia* Assemblage B isolate GS | [GenPept:EET01066] |
| *Giardia lamblia* Assemblage A2 isolate DH | [GenPept:EDO77667] |
| *Giardia lamblia* Assemblage B isolate GS_B | [GenPept:ESU45427] |
| *Giardia lamblia* Assemblage E isolate P15 | [GenPept:EFO62905] |
