# Supplementary material for: Variation in the ribosome interacting loop of the Sec61α from Giardia lamblia
Source: Biol Direct. 2015 Sep 30;10:56. doi: 10.1186/s13062-015-0087-0 (PMC4588681; doi:10.1186/s13062-015-0087-0)
Supplement: Additional file 6: — MSA of Sec61α sequences. (PDF 188 kb) [file 13062_2015_87_MOESM6_ESM.pdf]

CLUSTAL multiple sequence alignment by MUSCLE (3.8)

```
Giardia_Assem.A_WB         -----MLQFVKSQKRKTRGRGARMVATWLKVLDPWARMLPSVEKAPKT-VRFTTKGLYTF
Saccharomyces              ----------------------MSSNRVLDLFKPFESFLPEVIAPERK-VPYNQKLIWTG
oryza                      -----------------MAGRSSSSFRLLDLVRPFMPLLPEVREPDGRRVPFRRKLACTA
Fonticula                  -------------------------MRVLHLLRPVYSYLPEVKEPNKRAISFKEKLMWTG
Lygus                      ------------------------------------------------------------
Capsaspora                 MYSTSISSKDQSGFCFFTVSSFCQVMGFLHYLKPFISLIPEIEAPVKK-VPFKERAIYTV
Poecilia                   -----------------------MGIKFLEVIKPFCAVLPEIQKPERK-IQFREKVLWTA
Lygus2                     -----------------------MAIKFLEVIKPFCSILPEIAKPERK-IQFREKVLWTA
Chrysochromulina           --------------------MSTGKLRFLTLVKPVLHVLPEVAPPDRK-IPFKEKVLWTA
Brassica                   ---------------------MGGGFRVLHLVRPFLAFLPEVQSADRK-VPFREKVIYTV
Vitis                      ---------------------MGGGFRVLHLVRPFLSFLPEVQNADRK-IPFREKVIYTV
Aegilops                   ---------------------MAGGFRVLHLVRPFLGFLPEVQSADRR-IPFREKLIYTV
Triticum                   ---------------------MAGGFRVLHLVRPFLAFLPEVQSADRK-IPFREKVIYTV
Nematocida                 -------------------------MRILDFVKPFVPFLPEVQSSDRK-VLFQEKLLWTL
Spraguea                   --------------MTCKFLYFNFLVRTLDLIRPFVPFLPEIQSPGRQ-IPFQEKVVWTT
Trachipleistophora         -----------------------MAFRALSLIKPFVPFLPEVQLPGRE-INFQEKFVWTG
Ziziphus                   -------------------------------MTKLINFLVDKKK-------IINKIFFTL
Prunus                     -------------------------------MKMVIDFLRDKSK-------IIKKILFTL
Peptostreptococcaceae      ---------------------------------MFTALKNAFKIPD-----LKKKLVYTL
Pavlova                    ---------------------------------MKKAFVLE--------GPLVLRLFRTI
Porphyridium               ---------------------------------MKIS--------------LTKRIAITL
Pelagomonas                ---------------------------------MGLNFLGD-KSPSRD--NLVNRILLTL
Vaucheria                  ----------------------------------MNTKLQDSKSPR-----LKNRILLTI
Saccharina                 ---------------------------------MNLQLRSK-NTPS-----FRQRFFLTI


Giardia_Assem.A_WB         LALIVYLTASQVPLYGL-----------HSSIRKDPL----FWLRSIFAGQRGSL--MEL
Saccharomyces              VSLLIFLILGQIPLYGI-----------VSSETSDPL----YWLRAMLASNRGTL--LEL
oryza                      AALFAFLACSQLPLYGLHRAA-------AAGGGADPF----YWVRAILASNRGTV--MEL
Fonticula                  VCLLIYLVCSQVPLFGI-----------LSNEKADPF----YWARMILASNRGTL--MEL
Lygus                      -------------------------------------------MRVILASNRGTL--MEL
Capsaspora                 ITLVIFLVCCQVPLYGI-----------MSSDSADPF----FWMRAILASNRGTL--MEL
Poecilia                   ITLFIFLVCCQIPLFGI-----------MSSDSADPF----YWMRVILASNRGTL--MEL
Lygus2                     ITLFIFLVCCQIPLFGI-----------MSSDSADPF----YWIRVILASNRGTL--MEL
Chrysochromulina           ITLFIFLVCCQIPIYGC-----------KTNKSSDPF----YWMRVLLASNRGTL--MEL
Brassica                   ISLFIFLVCSQLPLYGI-----------HSTTGADPF----YWMRVILASNRGTV--MEL
Vitis                      ISLFIFLVCSQLPLYGI-----------HSTTGADPF----YWMRVILASSRGTV--MEL
Aegilops                   ISLFIFLVCSQLPLYGI-----------HSTTGADPF----YWLRAILASNRGTV--MEL
Triticum                   ISLFIFLVCSQLPLYGI-----------HSTTGADPF----YWMRVILASNRGTV--MEL
Nematocida                 IAILIYFVASQIPLFGI-----------MINDKADPV----YWLRAMMAGNRGTL--MDL
Spraguea                   VTLLIYLVSSQVPLYGI-----------ITANVKDPL----GWMRMMMASNRGTL--MDL
Trachipleistophora         VALLIYLVSSQVPLFGI-----------LNTNSADPF----YWMRMMMASNRGTL--MDL
Ziziphus                   FIIFIYVIGTSIYIPFL-SKSEYQ----AISVLRQLG----SSFNKSLLSNNNSLCILSL
Prunus                     VILFIFVIGNKLVIPSV------------TVQEKFLFDIKQYFSKSSLFEANGSIYFLSL
Peptostreptococcaceae      LMLVVFRVGSAIPVPGINVEVIRQ----MLQQYTSFS----SFYDLVSGGALNNFTIFAL
Pavlova                    MILIFARLGNYIPIPGITEVESFY----ESSFRNTSI----YNLSALSGGSN-VISILTL
Porphyridium               FLLIIVRLGVFLPVPGI-DHDSFY----NSTKSNQLI----GFLNIFSGGAFSTVGIFAL
Pelagomonas                GIILFARLGTFIPLTGI-DQKYLY----TELQNSPIL----NFFSSFSQGDFFVLGPFTL
Vaucheria                  SLLTIVRIGSFIPVPYITKEVLVNLLSAENSSNNTFA----QLLNTFSGGGNSSFGLLSL
Saccharina                 GLLTLVRIGSFIPLPYI-DIKTFSPLLESNTSTTAVA----TILNSFSGGENASFSILSL
                                                                                 .  : *

Giardia_Assem.A_WB         GIGPTITSSFFLKILISSKVLPFDKNNEVE-----SQVFNRVQNLIGFIFTFFQAVLYVL
Saccharomyces              GVSPIITSSMIFQFLQGTQLLQIRPESKQD-----RELFQIAQKVCAIILILGQALVVVM
oryza                      GITPVVTAGTLVQLLVGSNLVRADSSNPDD-----RALLSAAQKLLSIVITAGEATAYVL
Fonticula                  GITPLVTSGMILQVLVGANIIEVDQSVEED-----RELYAAAQKFIGLVWSLGQAIVSVY
Lygus                      GISPIVTASMVMQLLAGSHIIQVDQGLKED-----RILFQSAQKVLGLIITFSEAVAYVL
Capsaspora                 GISPIVTSGMIMQLLAGAKLLEVDQSNADE-----RALFSGAQRLFGLIITVGQAIIYVA
Poecilia                   GISPIVTSGLIMQLLAGAKIIEVGDTPKD------RALFNGAQKLFGMIITIGQAIVYVM
Lygus2                     GISPIVTSGLIMQLLAGAKIIEVGDTPKD------RALFNGAQKLFGMVITVGQAIVYVM
Chrysochromulina           GISPIITSGLVMQLLAGSKIIDVDQGLKED-----RALFSGAQKLFGILITVGEAVAYVI
Brassica                   GITPIVTSGLVMQLLAGSKIIEVDNNVRED-----RALLNGAQKLLGILIAIGEAVAYVL
Vitis                      GITPIVTSGLVMQLLAGSKIIEVDNNVRED-----RALLNGAQKLLGILIAVGEAVAYVL
Aegilops                   GITPIVTSGMVMQLLVGSKIIEVDNSVRED-----RALLNGAQKLLGILIAIGEAVAYVL
Triticum                   GITPIVTSGMVMQLLVGSKIIEVDNSVRED-----RALLNGAQKLLGILIAIGEAVAYVL
Nematocida                 GLGPILMTSSIIQFLGFVDLLKIDESIKED-----SLLMASLQKLLALIITFSQALVQIS
Spraguea                   GISPVITSGMILQMLTSSELIKVNFQLKED-----KLLYQALTKFLAVLLTVGQAIVQVI
Trachipleistophora         GISPVVTSSMIMQMLSSLEIVRVNSKVKED-----QTLYSAASKLLAVLLTVGQSIVQVS
Ziziphus                   GVIPYVTASIVIQLS--QKVFTFMKEWQEQ-GEKGKRKINIVTRVLTIFLSLGHGWALCQ
Prunus                     GVYPYITASIIVQFS--QKLFSFMKEWQEQ-GERGKYKTNLVARSLTLLFAVGLALSSIQ
Peptostreptococcaceae      GVGPYVTSSIIIQLL--AVAIPALEDMQKS-GEEGRKQIMQITRYTTVALALLQSTALGI
Pavlova                    GLGPFFSASLAVQFL--VKLYPAFEKLQNEEGEEGRKTIVRYTRILTVLFCIIESFFLSN
Porphyridium               GIVPYINASIIIQII--TKLIPNLRKLQEEEGEAGRNKITQITRILAFGWAVLQGLTISF
Pelagomonas                GILPNINASISMQLL--TAVLPFLQKLQKEEGVTGQKKVTQYTRYLTVVIAFIYGCLIAF
Vaucheria                  GILPYINASIIIQLL--TTIIPALSKMQKDEGEYGRRKLVDFTRYLTFFWAIVESISISY
Saccharina                 GILPNINASIIIQLL--TTINPTLLKLQKEEGEYGRRKLTDYTRYLTFFCAIIESIVTTY
```

```
                          *: * . :.  .:.                             .   .      .

Giardia_Assem.A_WB        AGIYGSISQIGLFSAVAIIAQLTISSILVQVLDEMLENGWGIGSGISLFTTANVC-----
Saccharomyces             TGNYGAPSDLGLPICLLLIFQLMFASLIVMLLDELLSKGYGLGSGISLFTATNIA-----
oryza                     SGAYGSVGVLGAGNAVLVVLQLVLGGMVAIFLDELLQKGYGFGSGISLFTAANTC-----
Fonticula                 TGMYGNPADLGLPICALLVLQLFLAGLLVMLIDELLSKGYGLGSGITLFISTNIC-----
Lygus                     SGIYGDIHDIGAMNALLIIAQLVIAGVVVLLLDELMQKGYGLGSGISLFLVTNIC-----
Capsaspora                TGLYGPVGELGFFVCFMLVLQLLIAGLIVMLLDELLQKGYGLGSGISLFIATNVC-----
Poecilia                  TGMYGDPSEMGAGICLLIIIQLFVAGLIVLLLDELLQKGYGLGSGISLFIATNIC-----
Lygus2                    TGMYGDPSEIGAGICLLIIVQLFVAGLIVLLLDELLQKGYGLGSGISLFIATNIC-----
Chrysochromulina          SGMYGDVRELGAGNSILIIILQLFVAGIIVIILDELLQKGYGLGSGISLFIATNIC-----
Brassica                  SGMYGPVGQLGVGNAILIIILQLFFAGIIVICLDELLQKGYGLGSGISLFIATNIC-----
Vitis                     SGMYGSVSQLGVGNAILIIVQLCFAGIIVICLDELLQKGYGLGSGISLFIATNIC-----
Aegilops                  SGMYGSVSQLGTGNAILIIILQLFFAGIIVICLDELLQKGYGLGSGISLFIATNICNVLAH
Triticum                  SGMYGSVSQLGTGNAILIIILQLFFAGIIVICLDELLQKGYGLGSGISLFIATNIC-----
Nematocida                TGFFGAPSNLGLSACILILVQLMFSGVIIILLDELLQKGYGLGSGVNLFIVANIC-----
Spraguea                  SGFYGSPNNLGLSVSVLLVVQLLFSGIIVIMLDELLQKGYGFGSGINLFIATNVC-----
Trachipleistophora        TGFYGPTSDIGIQVSLLLVIQLFFSGIIIILLDELLQKGYGLGSGVNLFIATNVC-----
Ziziphus                  T----EKFVLGPSLLFSTLFFLTVGVFISIWLADLITSK-GLGNGISILIAVGMV-----
Prunus                    RDGISSLAN-NKAHVLTIVFFLVVGAFISIWLADLITSK-GVGNGISIFVAISVS-----
Peptostreptococcaceae     GFFRSALVDNSFLNQASVVLTLTAGSAFLMWLGEQITEK-GIGNGISTLIFAGIV-----
Pavlova                   S-LRSFVFNWNSISYFVVAAAVTTGSLVLVWLSEVITER-GIGNGSSLLI---LI-----
Porphyridium              W-IRPYVFDWNLQFILDTTLGLCCGAMLEMWFAEIISEM-GIGNGTSILIFFNIV-----
Pelagomonas               F-LKPFVFGWNLVRAVEIALTLTTGSIIILWLSELITEK-GIGNGTSLFIFVNIS-----
Vaucheria                 S-LREVIFEWNLQVYFLISLSLITGSMIVLWFSELITKN-GLGNGSSLLICFNIV-----
Saccharina                S-FRPLIFNWNVLVLIQISLTLIAGSMIILWFSELITKD-GIGNGSSILICFNIV-----
                                    .          :  .  .   : : : .  *.*.* . :

Giardia_Assem.A_WB        -------ENIIWKSFSFFRIDRGNGKEFEGAVLAAVHYMFTQPNKLKAIKLAFFRDG---
Saccharomyces             -------EQIFWRAFAPTTVNSGRGKEFEGAVIAFFHLLAVRKDKKRALVEAFYRTN---
oryza                     -------EGVVTRALSPATMDRGRGAEFVGAVTAAAHLLATRARKLSAVREAFFRGGGGG
Fonticula                 -------ENILWKAFSPTTINLGRGTEFEGAIISLIYLLATRTDKTRALREAFYRDN---
Lygus                     -------ETIIWKAFSPVTITSGRGTEFEGAIIAFFHLLFTRSSKFNAIIEAFTRPH---
Capsaspora                -------ESIIWRSFSPTTVNTGRGTEFEGAVIGFFHLLATRSDKFKALREAFFRQN---
Poecilia                  -------ETIVWKAFSPTTVNTGRGTEFEGAIIALFHLLATRTDKVRALREAFYRQN---
Lygus2                    -------ETIVWKAFSPATVNTGRGTEFEGAVIALFHLLATRQDKVRALREAFYRQN---
Chrysochromulina          -------ETIVWKSFSPTTINTGRGTEFEGALVAFFHLVVSRPDKWKGVKEAFYRQN---
Brassica                  -------ESIIWKAFSPTTINTGRGAEFEGAVIALFHMLITKSNKVAALRQAFYRQN---
Vitis                     -------ENIIWKAFSPTTINSGRGAEFEGAVIALFHLLITRTDKVRALREAFYRQN---
Aegilops                  GKLVSYSENIIWKAFSPTTINSGRGAEFEGAVIGLFHLLITRTDKVRALREAFYRQN---
Triticum                  -------ENIIWKAFSPTTINSGRGAEFEGAVIALFHLLITRSDKVRALREAFYRQN---
Nematocida                -------ESIVWKAFSPSVYNTGKGPEFEGSLISLLQLLKIRRNKFEALYEAFFRKN---
Spraguea                  -------ENIVWKAFSPRVYKTARGIEFEGSIISLIYLLVTKKNKIAALYEAFFRQN---
Trachipleistophora        -------ESIVWKALSPKMHTTARGIEFEGSLIALFHLLFVRKNKLYALHEAFFREN---
Ziziphus                  -------DKLFKTFKYLLSNSNG----FETQRILILISYFVLLILTIILSSAYLK-----
Prunus                    -------KNLYDTLKFLTNVNN---PDFVAKRIFTLVVLLILLILTVILSAAYLK-----
Peptostreptococcaceae     -------SRVPSGAIKTFELLQTKELSIV-EFLLFMALYLAIIVGVIMVLEAVRK-----
Pavlova                   -------GNLSR--FRFLINKDDFDSLNVSSQSNLYIIYIIITLVSMLIFSTLSQEG---
Porphyridium              -------ANIPKNTITNWVLEGGFDFSFLQIAWMIIIIFFFMLAIAVILQEAVKK-----
Pelagomonas               -------SALPKTLNNFAVLVPTLPLK-----LGIGFIFAIGLLSIVVVQGALRK-----
Vaucheria                 -------SNLPDQIKFSLISLKNQINNFS-NIFLLISIFLITTIGCIYINEAIIK-----
Saccharina                -------SNFPDQLRAMILALSNQNIIVS---FFIGGIFLLTTVGCIYINEAMVK-----
                                   .                                      :  :  .

Giardia_Assem.A_WB        -LTNVMNIIATLVVFLVAIYLQGIKRNLRIQHAKAGPSVQ-QQYPIRLLYASSTPMMIIS
Saccharomyces             -LPNMFQVLMTVAIFLFVLYLQGFRYELPIRSTKVRGQI--GIYPIKLFYTSNTPIMLQS
oryza                     SLPDLRGLAATCAVFLAAVYLQGVRVALPVRPRNAPRSHRGGAYSVRLLYTSGMPVVLLS
Fonticula                 -LPNVTNLLATVLIFLIVIYFQGFRVDVRIQSTRGYSPP--MTYPIKLFYTSNMPIIFHS
Lygus                     -LPNLLNLCATIGIFLLVVYLQAFRIELPIASTKMSGQV--LPFSIKLFYTSNIPIILQT
Capsaspora                -LPNLTNLFATVAVFLIVIYFQGFRVDVPVVSRNAPGVV--QTYSIKLFYTSNMPIILQS
Poecilia                  -LPNLMNLIATVFVFAVVIYFQGFRVDLPIKSARYRGQY--NTYPIKLFYTSNIPIILQS
Lygus2                    -LPNLMNLLATILVFAIVIYFQGFRVDLPIKSARYRGQY--SSYPIKLFYTSNIPIILQS
Chrysochromulina          -LPNLTNLMSTVLIFLVVIYFQGFRVDLPVKYRSQRGMQ--GSYPIKLFYTSNMPIILQT
Brassica                  -LPNVTNLLATVLIFLIVIYFQGFRVVLPVRSKNARGQQ--GSYPIKLFYTSNMPIILQS
Vitis                     -LPNVTNLLATVLIFLIVIYFQGFRVVLPVRSKNARGQQ--GSYPIKLFYTSNMPIILQS
Aegilops                  -LPNVTNLLATVLVFLIVIYFQGFRVVLPVRSRNARGQQ--GSYPIKLFYTSNMPIILHS
Triticum                  -LPNVTNLLATVLVFLIVIYFQGFRVVLPVRSKNARGQQ--GSYPIKLFYTSNMPIILHS
Nematocida                -FPNISCLLTTVAMFSLVIYLYNIRLDLQLESTQMKTRY--INWGIKLFYVSSTPIIIQN
Spraguea                  -LPNCACFVSTIFIFCIVIYLQGLRVELPTESTQVRGQV--GRYPIKLLYASTMPIIVQN
Trachipleistophora        -LPNMMTLTSTLFIFALVIYVHGVRVNLRTESLQVKGQQ--GNYPIKLLYSSTMPIIVQN
Ziziphus                  ---------------IPINYAIN-------RNNDKIDKY----IPIKLNTSGILPIILAD
Prunus                    ---------------VPITYATK-------QNNDKIKNH----IPLKLNTSGILPVILAN
Peptostreptococcaceae     ---------------VQVQHAKRV-----IGRKTYGGQS--THIPIKINQAGVIPVIFAS
Pavlova                   ------------ARKIPVVSAKQL---IDGVEDDMRRSY----IPIRFGQAGVVPIIFSS
Porphyridium              ---------------ITIISAKQL---TEFGNSQTVSSY--SYIPLKLYQGGIMPIIFAS
Pelagomonas               ---------------IDLLSVKSLLR----ESSNSQSSY----LPFRLNPSGIMPLIFSS
Vaucheria                 ---------------IPLVSARQLLKKTKSEEKNSSNSI----LPLRINQAGVMPLVFTS
Saccharina                ---------------IPLVSASQL-----LRNVNKFSLWNNSNLPLRVNQAGVMPLVFTS
                                                                         ...   .  *::.

Giardia_Assem.A_WB        TLTSNVFMISQAIWRRFGNS---IFTALLGTWAEVESR-PGQAFPTGGLAWILASPYSLR
Saccharomyces             ALTSNIFLISQILFQKYPTN---PLIRLIGVWGIRPGT-QGPQMALSGLAYYIQPLMSLS
```

```
oryza                      SAVSSLYVVSQALYRRFGGS---LLVDLLGKWTPDAAV------PVGGIAYYVTAPASAA
Fonticula                  ALVTNVYLFSQILYNQFPEN---FLVRMFGVWRNHPGS--SQLFAVGGLAYYMSPPRTLA
Lygus                      ALVSNLYFFSQLLHKRYSNN---FLVSLLGVWEDTSSG---ISIPIGGLAYYVSPPTSLL
Capsaspora                 ALVQNLFIISQLLWFKLSHTGLGWIIGLLGSWENVAYQGSNRSYPVGGLCYYLSPPNGLT
Poecilia                   ALVSNLYVISQMLSTRFSGN---FLVNLLGTWSDTSSGGPARAYPVGGLCYYLSPPESFG
Lygus2                     ALVSNLYVISQMLAVKFHGN---VFVNLLGVWADVGGGGPARAYPVGGLCYYLSPPENLG
Chrysochromulina           ALVSNLYFLSQLMYKRFPNN---VLVCWFGRWKEDETAGPSGNAPVGGLVYYLSPPNSIA
Brassica                   ALVSNLYFISQLLYRKFSGN---FFVNLLGQWKESEYS--GQSIPVSGLAYLITAPASFS
Vitis                      ALVTNLYFISQLLYRRYSGN---FLVNLLGKWKESEYS-GGQYIPVGGLAYYITAPSSLA
Aegilops                   ALITNLYFISQLLYKKFSGN---FLVNLLGIWKESEYS--GHSIPVGGLAYYVTAPSSLA
Triticum                   ALITNLYFISQLLYRKYSGN---FLVNLLGIWKESEYS--GHSIPVGGLAYYVTAPSSMA
Nematocida                 QILTNYYRISKFLFDRFPTK---WYTRILGLWDINESM---IYVPVKGIAYFISPPVNIL
Spraguea                   YMVTYTSTASRLLYNKFPHF---FLVRLLGVWEHIKGG---NIVPVSGICYFLYPPQNLT
Trachipleistophora         YIISHASTVSRFLYQKFPDV---FLVRLLGVWTMRKNG---KMVPISGICYFLFPPDSLM
Ziziphus                   TLLNVIQQISMLFSKNG------KVNEYIGIFVESRSE--------LGI-YFF-------
Prunus                     SLLQVFGTISLLLGPEN------GFSQWVNRFQDSQSN-------YLGL-GFF-------
Peptostreptococcaceae      SFLMMPNLLGIFIKNQ-------SYQTFVSKYFSTQG-------PPGLYVYAL-------
Pavlova                    SILLFLTTSIKQLPNAN------IATRVILDSVN-----------LQQIFYFF-------
Porphyridium               AIIGLPTYVLQITNNP-------VLQEIF------------LQMSPKGIFFLP-------
Pelagomonas                GLLNVLIVGINKIS---------LLQGLAN---------------FSNLIYTS-------
Vaucheria                  YAILFFSSLFEIIKKQT------NIFNIFFQYPILNSV---ISYWFLKILFWI-------
Saccharina                 SVMVILSSLTNLIYGQL------VNIELFSFLNSLSTN---LTLGVGKIFYWS-------

Giardia_Assem.A_WB         SALFHPIHTILHAVTLVALSGLISRVWVEFSGEGAKEVAEMLE-----------------
Saccharomyces              EALLDPIKTIVYITFVLGSCAVFSKTWIEISGTSPRDIAKQFK-----------------
oryza                      SAAANPLHAAMYVAFVLAACAALSRAWVVVSGSSSRDVARRLR-----------------
Fonticula                  DIASDPVHTIIYIAFVLGTCALFSSIWVSFSGQSARDVAAQFR-----------------
Lygus                      DLVYDPIHSVVYIAFILVSCAIFSRSWIDVSGSSPKDVAKQLA-----------------
Capsaspora                 GVVADPLHGMIYIAFILGTCALFSLLWIDLSGASSQDVARQLR-----------------
Poecilia                   SVLDDPVHAVIYIVFMLGSCAFFSKTWIEVSGSSAKDVAKQLK-----------------
Lygus2                     HILEDPVHAVLYIIFMLGSCAFFSKTWIEVSGSSAKDVAKQLK-----------------
Chrysochromulina           EVVADPIHAVFYLMFILVACALFSKTWIEVSGSSAKDVAKQLR-----------------
Brassica                   DMAAHPFHALFYIVFMLTACALFSKTWIEVSGSSARDVAKQLK-----------------
Vitis                      DMAANPFHALFYLIFMLAACALFSKTWIEVSGSSARDVAKQLK-----------------
Aegilops                   DVVANPFHALFYVVFMLSACALFSKTWIEVSGSSARDVARQLKLVLHMNSHYARATRNLL
Triticum                   DILANPFHALFYVVFMLSACALFSKTWIEVSGSSAKDVAKQLK-----------------
Nematocida                 AALKNPIHFLIYTSFMLTTSGLLAYYWVDMNESSPKEVGKQLQ-----------------
Spraguea                   EFITKPIHSIIYVSFILLFGAFFSRAWIDITENNQNSVAEQMK-----------------
Trachipleistophora         DIFRKPLYFMVYTSIVLLSSAFLSRAWIDMTESNQNDVARSLI-----------------
Ziziphus                   ----------VYILLIMLFSFFSSFMTIN-----PKDVAEHLS-----------------
Prunus                     ----------VYLLLILLFSVFSTFITIN-----PTDIAEHLS-----------------
Peptostreptococcaceae      ----------LEFLLVVGFSYFYVEIIFK-----PDEIADNLK-----------------
Pavlova                    ----------TFLVLIIFFSFFYTLIILS-----PSDIAKNLK-----------------
Porphyridium               ----------LYYLLILSFSFIYTSLILN-----PEDISKNLR-----------------
Pelagomonas                ----------GYFILTLFFSYFYSTIAIK-----PSDLSDNLK-----------------
Vaucheria                  ----------FYATLIFFFTYFYSTIVLD-----PKDVAERFR-----------------
Saccharina                 ----------TYGILVFFFTSFYSTILLD-----PKDMTEQFR-----------------
                                          .         .       .:   :

Giardia_Assem.A_WB         ------TNGWCMPGYMTK-GALQRELNRYIPTAALAGGLILGFVGFCADIFGAI------
Saccharomyces              ------DQGMVINGKRET--SIYRELKKIIPTAAAFGGATIGALSVGSDLLGTL------
oryza                      ------EQQMVMPGMREA--SMQRELERYIPAAAALGGVCVGALTVAADLMGAV------
Fonticula                  ------AQSITIKGHSN---NVEGFLNRFIPIAAAFGGLCIGALSIFADFTGAI------
Lygus                      ------DQDRLIKGFPPN--QTERVLSYYIPIAAAFGGMCIGALTLVADLFNAI------
Capsaspora                 ------EQQMFVKGHKDTQESTARQLNRYIPTAAAFGGLCIGALSITADFFGAI------
Poecilia                   ------EQQMVMRGHRET--SMVHELNRYIPTAAAFGGLCIGGLSVMADFLGAI------
Lygus2                     ------EQQMVMRGHRDN--SMIHELNRYIPTAAAFGGLCIGALSVLADFMGAI------
Chrysochromulina           ------DQQMVMKGHRDS--SLAKELNRYIPTAAAFGGMCIGALTVIADFMGAI------
Brassica                   ------EQQMVMPGHRES--NLQKELNRYIPTAAAFGGVCIGALTVLADFMGAI------
Vitis                      ------EQQMVMPGHREA--NLQKELNRYIPTAAAFGGMCIGALTVLADFMGAI------
Aegilops                   TLATEQEQQMVMPGHRES--NLERELNRYIPTAAAFGGVCIGALTVLADFMGAI------
Triticum                   ------EQQMVMPGHRES--NLQKELNKYIPTAAAFGGVCIGALTVLADFMGAI------
Nematocida                 ------KQKLVVKGYSVQ--GTQDMLDRYIPIAAVLSGLIVGGISIMSDLLDTI------
Spraguea                   ------SQKITLRGVKQD--NIAHHLEKYVPTAAFLSGFFVGLVVLLSDLLDTI------
Trachipleistophora         ------NRRVTIKGVPER--NLANKLGEYIPTAAFLGGLVIGFIVMLSNILDTI------
Ziziphus                   ------KQNAYLKDVQPG-LPTVKKIVREMFKITFLGSCFLTLLASTPDIINYL---AGS
Prunus                     ------KQDAYLEGVKPG-DETVYKITQKLFKVTVIGAFALTVLAALPEFIKFFVWTNDS
Peptostreptococcaceae      ------NSAGFIPGIKPG-RNTAEYLQEISNRLTLAGALFLAAISAIPILILGF------
Pavlova                    ------KMSSVIQDTKPG-VATKVYIRKFILQASFVGSILLSALILIPSILAAA------
Porphyridium               ------KTGSSIVGVKPG-ADTVKYLQTTLNRLTFLGSILLFLIAVMPVFVSFF------
Pelagomonas                ------KMNFTIPGVQPG-LATMRFLQETLTRLALLGGLFLAIIVTIPSLLAFV------
Vaucheria                  ------KNSVVILGISPG-SSTRSYLSKILRFIAKINAIFLIYNIIGLQILESI----LN
Saccharina                 ------KNSVTIKGITPG-KSTQSYLSITIKRLTILNAVFLIGVLILLNGLEYL----LP
                                      : .           :       :  ..  :

Giardia_Assem.A_WB         -------GSG-TGILLAATTLVKMYEEFAKEGIQLSM---------
Saccharomyces              -------GSG-ASILMATTTIYGYYEAAAKEGGFTKNLVPGFSDLM
```

```
oryza                     -------GSG-AGMLLAVTTVYQCYEAFEKEKTY-----------
Fonticula                 -------GSG-TGILLAVSSIFQYYELFVKEQREKGVSLQSLME--
Lygus                     -------GSG-TGILLAVTIIYQYFELYAREQFTM-----------
Capsaspora                -------GSG-TGILMAVTTIYQYYEIMAKEQISMGNLF-------
Poecilia                  -------GSG-TGILLAVTIIYQYFEIFVKEQSEVGSMGALLF---
Lygus2                    -------GSG-TGILLAVTIIYQYFEIFVKEQSEMGGMSTLLF---
Chrysochromulina          -------GSG-TGILLAVTTIYQYFEMFVKENGQDNALSVFF----
Brassica                  -------GSG-TGILLAVTIIYQYFETFEKEKASELGFFGF-----
Vitis                     -------GSG-TGILLAVTIIYQYFETFEKERASELGFFGF-----
Aegilops                  -------GSG-TGILLAVTIIYQYFETFEKERATELGFFGF-----
Triticum                  -------GSG-TGILLADSIIYQYFETFEKERATELGFFGF-----
Nematocida                -------GSG-QNIILAVSIIGQYFELFVKEQMKYKGMQ-------
Spraguea                  -------GSG-TNIILAVSIVWQYLELFVKESLTMKGMAFID----
Trachipleistophora        -------GSG-TNIFLAVSIVWQYCELFNKEAAKRGGLLVVD----
Ziziphus                  TISQNIPFGG-TSLLIIVGVALESIQDMKA----------------
Prunus                    DKTFKVQLGG-TSLLIVVGVAVEVMQRITTQTNVKKLYKKLF----
Peptostreptococcaceae     -TSIPFRFGG-TSLLIVVGVALETMKQIEAQMVMRHYQGFLK----
Pavlova                   LGVHPLSISGITSLILSFSIINDTVRQVLAYRDTRKFLLSS-----
Porphyridium              -KIDILKTVNPTSLLILVGVAIQTTKQIQVYILSKEFESLTSKEK-
Pelagomonas               --NPAIKGFGITSLIILVGVAVDLSRQIRFYLISEVYDNINL----
Vaucheria                 LNIINIRGLGFTSQLILVNVLIDTIKRIRSFLNEEENYF-------
Saccharina                VNNLNLRGFGLTSQLILVNVLVDTFRKVRNLLNAETMF--------
                                   .  . ::
```

Capsaspora owczarzaki

```
>gi|765556507|gb|KJE96835.1| Sec61a1 protein [Capsaspora owczarzaki ATCC 30864]
MYSTSISSKDQSGFCFFTVSSFCQVMGFLHYLKPFISLIPEIEAPVKKVPFKERAIYTVITLVIFLVCCQ
VPLYGIMSSDSADPFFWMRAILASNRGTLMELGISPIVTSGMIMQLLAGAKLLEVDQSNADERALFSGAQ
RLFGLIITVGQAIIYVATGLYGPVGELGFFVCFMLVLQLLIAGLIVMLLDELLQKGYGLGSGISLFIATN
VCESIIWRSFSPTTVNTGRGTEFEGAVIGFFHLLATRSDKFKALREAFFRQNLPNLTNLFATVAVFLIVI
YFQGFRVDVPVVSRNAPGVVQTYSIKLFYTSNMPIILQSALVQNLFIISQLLWFKLSHTGLGWIIGLLGS
WENVAYQGSNRSYPVGGLCYYLSPPNGLTGVVADPLHGMIYIAFILGTCALFSLLWIDLSGASSQDVARQ
LREQQMFVKGHKDTQESTARQLNRYIPTAAAFGGLCIGALSITADFFGAIGSGTGILMAVTTIYQYYEIM
AKEQISMGNLF
```

Spraguea lophii

```
>gi|523779734|gb|EPR79257.1| SEC61 alpha subunit [Spraguea lophii 42_110]
MTCKFLYFNFLVRTLDLIRPFVPFLPEIQSPGRQIPFQEKVVWTTVTLLIYLVSSQVPLFGIITANVKDP
LGWMRMMMASNRGTLMDLGISPVITSGMILQMLTSSELIKVNFQLKEDKLLYQALTKFLAVLLTVGQAIV
QVISGFYGSPNNLGLSVSVLLVVQLLFSGIIVIMLDELLQKGYGFGSGINLFIATNVCENIVWKAFSPRV
YKTARGIEFEGSIISLIYLLVTKKNKIAALYEAFFRQNLPNCACFVSTIFIFCIVIYLQGLRVELPTEST
QVRGQVGRYPIKLLYASTMPIIVQNYMVTYTSTASRLLYNKFPHFFLVRLLGVWEHIKGGNIVPVSGICY
FLYPPQNLTEFITKPIHSIIYVSFILLFGAFFSRAWIDITENNQNSVAEQMKSQKITLRGVKQDNIAHHL
EKYVPTAAFLSGFFVGLVVLLSDLLDTIGSGTNIILAVSIVWQYLELFVKESLTMKGMAFID
```

Peptostreptococcaceae bacterium

```
>gi|402274364|gb|EJU23548.1| preprotein translocase, SecY subunit [Peptostreptococcaceae
bacterium OBRC8]
MFTALKNAFKIPDLKKKLVYTLLMLVVFRVGSAIPVPGINVEVIRQMLQQYTSFSSFYDLVSGGALNNFT
IFALGVGPYVTSSIIIQLLAVAIPALEDMQKSGEEGRKQIMQITRYTTVALALLQSTALGIGFFRSALVD
NSFLNQASVVLTLTAGSAFLMWLGEQITEKGIGNGISTLIFAGIVSRVPSGAIKTFELLQTKELSIVEFL
LFMALYLAIIVGVIMVLEAVRKVQVQHAKRVIGRKTYGGQSTHIPIKINQAGVIPVIFASSFLMMPNLLG
IFIKNQSYQTFVSKYFSTQGPPGLYVYALLEFLLVVGFSYFYVEIIFKPDEIADNLKNSAGFIPGIKPGR
NTAEYLQEISNRLTLAGALFLAAISAIPILILGFTSIPFRFGGTSLLIVVGVALETMKQIEAQMVMRHYQ
GFLK
```

Poecilia formosa

```
>gi|617435911|ref|XP_007563430.1| PREDICTED: protein transport protein Sec61 subunit alpha-
like 1 [Poecilia formosa]
MGIKFLEVIKPFCAVLPEIQKPERKIQFREKVLWTAITLFIFLVCCQIPLFGIMSSDSADPFYWMRVILA
SNRGTLMELGISPIVTSGLIMQLLAGAKIIEVGDTPKDRALFNGAQKLFGMIITIGQAIVYVMTGMYGDP
```

SEMGAGICLLIIIQLFVAGLIVLLLDELLQKGYGLGSGISLFIATNICETIVWKAFSPTTVNTGRGTEFE
GAIIALFHLLATRTDKVRALREAFYRQNLPNLMNLIATVFVFAVVIYFQGFRVDLPIKSARYRGQYNTYP
IKLFYTSNIPIILQSALVSNLYVISQMLSTRFSGNFLVNLLGTWSDTSSGGPARAYPVGGLCYYLSPPES
FGSVLDDPVHAVIYIVFMLGSCAFFSKTWIEVSGSSAKDVAKQLKEQQMVMRGHRETSMVHELNRYIPTA
AAFGGLCIGGLSVMADFLGAIGSGTGILLAVTIIYQYFEIFVKEQSEVGSMGALLF

Fonticula alba

>gi|627946594|gb|KCV68588.1| protein transporter SEC61 subunit alpha [Fonticula alba]
MRVLHLLRPVYSYLPEVKEPNKRAISFKEKLMWTGVCLLIYLVCSQVPLFGILSNEKADPFYWARMILAS
NRGTLMELGITPLVTSGMILQVLVGANIIEVDQSVEEDRELYAAAQKFIGLVWSLGQAIVSVYTGMYGNP
ADLGLPICALLVLQLFLAGLLVMLIDELLSKGYGLGSGITLFISTNICENILWKAFSPTTINLGRGTEFE
GAIISLIYLLATRTDKTRALREAFYRDNLPNVTNLLATVLIFLIVIYFQGFRVDVRIQSTRGYSPPMTYP
IKLFYTSNMPIIFHSALVTNVYLFSQILYNQFPENFLVRMFGVWRNHPGSSQLFAVGGLAYYMSPPRTLA
DIASDPVHTIIYIAFVLGTCALFSSIWVSFSGQSARDVAAQFRAQSITIKGHSNNVEGFLNRFIPIAAAF
GGLCIGALSIFADFTGAIGSGTGILLAVSSIFQYYELFVKEQREKGVSLQSLME

Lygus hesperus

>gi|732655856|gb|JAG41434.1| Protein transport protein Sec61 subunit alpha isoform 2 [Lygus hesperus]
MAIKFLEVIKPFCSILPEIAKPERKIQFREKVLWTAITLFIFLVCCQIPLFGIMSSDSADPFYWIRVILA
SNRGTLMELGISPIVTSGLIMQLLAGAKIIEVGDTPKDRALFNGAQKLFGMVITVGQAIVYVMTGMYGDP
SEIGAGICLLIIVQLFVAGLIVLLLDELLQKGYGLGSGISLFIATNICETIVWKAFSPATVNTGRGTEFE
GAVIALFHLLATRQDKVRALREAFYRQNLPNLMNLLATILVFAIVIYFQGFRVDLPIKSARYRGQYSSYP
IKLFYTSNIPIILQSALVSNLYVISQMLAVKFHGNVFVNLLGVWADVGGGGPARAYPVGGLCYYLSPPEN
LGHILEDPVHAVLYIIFMLGSCAFFSKTWIEVSGSSAKDVAKQLKEQQMVMRGHRDNSMIHELNRYIPTA
AAFGGLCIGALSVLADFMGAIGSGTGILLAVTIIYQYFEIFVKEQSEMGGMSTLLF

>gi|732599543|gb|JAG15195.1| Protein transport protein Sec61 subunit alpha [Lygus hesperus]
MRVILASNRGTLMELGISPIVTASMVMQLLAGSHIIQVDQGLKEDRILFQSAQKVLGLIITFSEAVAYVL
SGIYGDIHDIGAMNALLIIAQLVIAGVVVLLLDELMQKGYGLGSGISLFLVTNICETIIWKAFSPVTITS
GRGTEFEGAIIAFFHLLFTRSSKFNAIIEAFTRPHLPNLLNLCATIGIFLLVVYLQAFRIELPIASTKMS
GQVLPFSIKLFYTSNIPIILQTALVSNLYFFSQLLHKRYSNNFLVSLLGVWEDTSSGISIPIGGLAYYVS
PPTSLLDLVYDPIHSVVYIAFILVSCAIFSRSWIDVSGSSPKDVAKQLADQDRLIKGFPPNQTERVLSYY
IPIAAAFGGMCIGALTLVADLFNAIGSGTGILLAVTIIYQYFELYAREQFTM

Trachipleistophora hominis

>gi|440492013|gb|ELQ74615.1| Transport protein Sec61, alpha subunit [Trachipleistophora hominis]
MAFRALSLIKPFVPFLPEVQLPGREINFQEKFVWTGVALLIYLVSSQVPLFGILNTNSADPFYWMRMMMA
SNRGTLMDLGISPVVTSSMIMQMLSSLEIVRVNSKVKEDQTLYSAASKLLAVLLTVGQSIVQVSTGFYGP
TSDIGIQVSLLLVIQLFFSGIIIILLDELLQKGYGLGSGVNLFIATNVCESIVWKALSPKMHTTARGIEF
EGSLIALFHLLFVRKNKLYALHEAFFRENLPNMMTLTSTLFIFALVIYVHGVRVNLRTESLQVKGQQGNY
PIKLLYSSTMPIIVQNYIISHASTVSRFLYQKFPDVFLVRLLGVWTMRKNGKMVPISGICYFLFPPDSLM
DIFRKPLYFMVYTSIVLLSSAFLSRAWIDMTESNQNDVARSLINRRVTIKGVPERNLANKLGEYIPTAAF
LGGLVIGFIVMLSNILDTIGSGTNIFLAVSIVWQYCELFNKEAAKRGGLLVVD

Nematocida parisii:

>gi|387595255|gb|EIJ92880.1| preprotein translocase [Nematocida parisii ERTm1]
MRILDFVKPFVPFLPEVQSSDRKVLFQEKLLWTLIAILIYFVASQIPLFGIMINDKADPVYWLRAMMAGN
RGTLMDLGLGPILMTSSIIQFLGFVDLLKIDESIKEDSLLMASLQKLLALIITFSQALVQISTGFFGAPS
NLGLSACILILVQLMFSGVIIILLDELLQKGYGLGSGVNLFIVANICESIVWKAFSPSVYNTGKGPEFEG
SLISLLQLLKIRRNKFEALYEAFFRKNFPNISCLLTTVAMFSLVIYLYNIRLDLQLESTQMKTRYINWGI

KLFYVSSTPIIIQNQILTNYYRISKFLFDRFPTKWYTRILGLWDINESMIYVPVKGIAYFISPPVNILAA
LKNPIHFLIYTSFMLTTSGLLAYYWVDMNESSPKEVGKQLQKQKLVVKGYSVQGTQDMLDRYIPIAAVLS
GLIVGGISIMSDLLDTIGSGQNIILAVSIIGQYFELFVKEQMKYKGMQ

Brassica oleracea

>gi|922478016|ref|XP_013637288.1| PREDICTED: protein transport protein Sec61 subunit alpha [Brassica oleracea var. oleracea]
MGGGFRVLHLVRPFLAFLPEVQSADRKVPFREKVIYTVISLFIFLVCSQLPLYGIHSTTGADPFYWMRVI
LASNRGTVMELGITPIVTSGLVMQLLAGSKIIEVDNNVREDRALLNGAQKLLGILIAIGEAVAYVLSGMY
GPVGQLGVGNAILIILQLFFAGIIVICLDELLQKGYGLGSGISLFIATNICESIIWKAFSPTTINTGRGA
EFEGAVIALFHMLITKSNKVAALRQAFYRQNLPNVTNLLATVLIFLIVIYFQGFRVVLPVRSKNARGQQG
SYPIKLFYTSNMPIILQSALVSNLYFISQLLYRKFSGNFFVNLLGQWKESEYSGQSIPVSGLAYLITAPA
SFSDMAAHPFHALFYIVFMLTACALFSKTWIEVSGSSARDVAKQLKEQQMVMPGHRESNLQKELNRYIPT
AAAFGGVCIGALTVLADFMGAIGSGTGILLAVTIIYQYFETFEKEKASELGFFGF

Aegilops tauschii

>gi|475625840|gb|EMT33101.1| Protein transport protein Sec61 subunit alpha [Aegilops tauschii]
MAGGFRVLHLVRPFLGFLPEVQSADRRIPFREKLIYTVISLFIFLVCSQLPLYGIHSTTGADPFYWLRAI
LASNRGTVMELGITPIVTSGMVMQLLVGSKIIEVDNSVREDRALLNGAQKLLGILIAIGEAVAYVLSGMY
GSVSQLGTGNAILIIILQLFFAGIIVICLDELLQKGYGLGSGISLFIATNICNVLAHGKLVSYSENIIWKA
FSPTTINSGRGAEFEGAVIGLFHLLITRTDKVRALREAFYRQNLPNVTNLLATVLVFLIVIYFQGFRVVL
PVRSRNARGQQGSYPIKLFYTSNMPIILHSALITNLYFISQLLYKKFSGNFLVNLLGIWKESEYSGHSIP
VGGLAYYVTAPSSLADVVANPFHALFYVVFMLSACALFSKTWIEVSGSSARDVARQLKLVLHMNSHYARA
TRNLLTLATEQEQQMVMPGHRESNLERELNRYIPTAAAFGGVCIGALTVLADFMGAIGSGTGILLAVTII
YQYFETFEKERATELGFFGF

Ziziphus jujuba

>gi|31075967|gb|AAP42343.1| translocation protein secY, partial [Jujube witches'-broom phytoplasma]
MTKLINFLVDKKKIINKIFFTLFIIFIYVIGTSIYIPFLSKSEYQAISVLRQLGSSFNKSLLSNNNSLCI
LSLGVIPYVTASIVIQLSQKVFTFMKEWQEQGEKGKRKINIVTRVLTIFLSLGHGWALCQTEKFVLGPSL
LFSTLFFLTVGVFISIWLADLITSKGLGNGISILIAVGMVDKLFKTFKYLLSNSNGFETQRILILISYFV
LLILTIILSSAYLKIPINYAINRNNDKIDKYIPIKLNTSGILPIILADTLLNVIQQISMLFSKNGKVNEY
IGIFVESRSELGIYFFVYILLIMLFSFFSSFMTINPKDVAEHLSKQNAYLKDVQPGLPTVKKIVREMFKI
TFLGSCFLTLLASTPDIINYLAGSTISQNIPFGGTSLLIIVGVALESIQDMKA

Pavlova lutheri

>gi|12120|emb|CAA45998.1| secY (chloroplast) [Pavlova lutheri]
MKKAFVLEGPLVLRLFRTIMILIFARLGNYIPIPGITEVESFYESSFRNTSIYNLSALSGGSNVISILTL
GLGPFFSASLAVQFLVKLYPAFEKLQNEEGEEGRKTIVRYTRILTVLFCIIESFFLSNSLRSFVFNWNSI
SYFVVAAAVTTGSLVLVWLSEVITERGIGNGSSLLILIGNLSRFRFLINKDDFDSLNVSSQSNLYIIYII
ITLVSMLIFSTLSQEGARKIPVVSAKQLIDGVEDDMRRSYIPIRFGQAGVVPIIFSSSILLFLTTSIKQL
PNANIATRVILDSVNLQQIFYFFTFLVLIIFFSFFYTLIILSPSDIAKNLKKMSSVIQDTKPGVATKVYI
RKFILQASFVGSILLSALILIPSILAAALGVHPLSISGITSLILSFSIINDTVRQVLAYRDTRKFLLSS

Chrysochromulina sp. CCMP291

>gi|922864600|gb|KOO31825.1| protein transport protein sec61 alpha subunit [Chrysochromulina sp. CCMP291]
MSTGKLRFLTLVKPVLHVLPEVAPPDRKIPFKEKVLWTAITLFIFLVCCQIPIYGCKTNKSSDPFYWMRV
LLASNRGTLMELGISPIITSGLVMQLLAGSKIIDVDQGLKEDRALFSGAQKLFGILITVGEAVAYVISGM
YGDVRELGAGNSILIILQLFVAGIIVIILDELLQKGYGLGSGISLFIATNICETIVWKSFSPTTINTGRG

TEFEGALVAFFHLVVSRPDKWKGVKEAFYRQNLPNLTNLMSTVLIFLVVIYFQGFRVDLPVKYRSQRGMQ
GSYPIKLFYTSNMPIILQTALVSNLYFLSQLMYKRFPNNVLVCWFGRWKEDETAGPSGNAPVGGLVYYLS
PPNSIAEVVADPIHAVFYLMFILVACALFSKTWIEVSGSSAKDVAKQLRDQQMVMKGHRDSSLAKELNRY
IPTAAAFGGMCIGALTVIADFMGAIGSGTGILLAVTTIYQYFEMFVKENGQDNALSVFF

Vaucheria litorea

>gi|215400755|ref|YP_002327516.1| preprotein-translocase subunit Y (chloroplast) [Vaucheria litorea]
MNTKLQDSKSPRLKNRILLTISLLTIVRIGSFIPVPYITKEVLVNLLSAENSSNNTFAQLLNTFSGGGNS
SFGLLSLGILPYINASIIIQLLTTIIPALSKMQKDEGEYGRRKLVDFTRYLTFFWAIVESISISYSLREV
IFEWNLQVYFLISLSLITGSMIVLWFSELITKNGLGNGSSLLICFNIVSNLPDQIKFSLISLKNQINNFS
NIFLLISIFLITTIGCIYINEAIIKIPLVSARQLLKKTKSEEKNSSNSILPLRINQAGVMPLVFTSYAIL
FFSSLFEIIKKQTNIFNIFFQYPILNSVISYWFLKILFWIFYATLIFFFTYFYSTIVLDPKDVAERFRKN
SVVILGISPGSSTRSYLSKILRFIAKINAIFLIYNIIGLQILESILNLNIININIRGLGFTSQLILVNVLID
TIKRIRSFLNEEENYF

Porphyridium purpureum

>gi|568247761|ref|YP_008965804.1| preprotein translocase secY subunit (chloroplast) [Porphyridium purpureum]
MKISLTKRIAITLFLLIIVRLGVFLPVPGIDHDSFYNSTKSNQLIGFLNIFSGGAFSTVGIFALGIVPYI
NASIIIQIITKLIPNLRKLQEEEGEAGRNKITQITRILAFGWAVLQGLTISFWIRPYVFDWNLQFILDTT
LGLCCGAMLEMWFAEIISEMGIGNGTSILIFFNIVANIPKNTITNWVLEGGFDFSFLQIAWMIIIIFFFM
LAIAVILQEAVKKITIISAKQLTEFGNSQTVSSYSYIPLKLYQGGIMPIIFASAIIGLPTYVLQITNNPV
LQEIFLQMSPKGIFFLPLYYLLILSFSFIYTSLILNPEDISKNLRKTGSSIVGVKPGADTVKYLQTTLNR
LTFLGSILLFLIAVMPVFVSFFKIDILKTVNPTSLLILVGVAIQTTKQIQVYILSKEFESLTSKEK

Pelagomonas

>gi|403225195|gb|AFR24807.1| preprotein translocase subunit (plastid) [uncultured Pelagomonas]
MGLNFLGDKSPSRDNLVNRILLTLGIILFARLGTFIPLTGIDQKYLYTELQNSPILNFFSSFSQGDFFVL
GPFTLGILPNINASISMQLLTAVLPFLQKLQKEEGVTGQKKVTQYTRYLTVVIAFIYGCLIAFFLKPFVF
GWNLVRAVEIALTLTTGSIIILWLSELITEKGIGNGTSLFIFVNISSALPKTLNNFAVLVPTLPLKLGIG
FIFAIGLLSIVVVQGALRKIDLLSVKSLLRESSNSQSSYLPFRLNPSGIMPLIFSSGLLNVLIVGINKIS
LLQGLANFSNLIYTSGYFILTLFFSYFYSTIAIKPSDLSDNLKKMNFTIPGVQPGLATMRFLQETLTRLA
LLGGLFLAIIVTIPSLLAFVNPAIKGFGITSLIILVGVAVDLSRQIRFYLISEVYDNINL

Saccharina japonica

>gi|403066618|ref|YP_006639107.1| preprotein translocase subunit secY (chloroplast) [Saccharina japonica]
MNLQLRSKNTPSFRQRFFLTIGLLTLVRIGSFIPLPYIDIKTFSPLLESNTSTTAVATILNSFSGGENAS
FSILSLGILPNINASIIIQLLTTINPTLLKLQKEEGEYGRRKLTDYTRYLTFFCAIIESIVTTYSFRPLI
FNWNVLVLIQISLTLIAGSMIILWFSELITKDGIGNGSSILICFNIVSNFPDQLRAMILALSNQNIIVSF
FIGGIFLLTTVGCIYINEAMVKIPLVSASQLLRNVNKFSLWNNSNLPLRVNQAGVMPLVFTSSVMVILSS
LTNLIYGQLVNIELFSFLNSLSTNLTLGVGKIFYWSTYGILVFFFTSFYSTILLDPKDMTEQFRKNSVTI
KGITPGKSTQSYLSITIKRLTILNAVFLIGVLILLNGLEYLLPVNNLNLRGFGLTSQLILVNVLVDTFRK
VRNLLNAETMF

Capsaspora polysiphoniae: no items found

Prunus persica

>gi|373943350|gb|AEY80145.1| SecY protein translocase ['Prunus persica' x-disease phytoplasma]
MKMVIDFLRDKSKIIKKILFTLVILFIFVIGNKLVIPSVTVQEKFLFDIKQYFSKSSLFEANGSIYFLSL
GVYPYITASIIVQFSQKLFSFMKEWQEQGERGKYKTNLVARSLTLLFAVGLALSSIQRDGISSLANNKAH

VLTIVFFLVVGAFISIWLADLITSKGVGNGISIFVAISVSKNLYDTLKFLTNVNNPDFVAKRIFTLVVLL
ILLILTVILSAAYLKVPITYATKQNNDKIKNHIPLKLNTSGILPVILANSLLQVFGTISLLLGPENGFSQ
WVNRFQDSQSNYLGLGFFVYLLLILLFSVFSTFITINPTDIAEHLSKQDAYLEGVKPGDETVYKITQKLF
KVTVIGAFALTVLAALPEFIKFFVWTNDSDKTFKVQLGGTSLLIVVGVAVEVMQRITTQTNVKKLYKKLF

***Volvox carteri*: No items found**

***Selaginella moellendorffi*i: No items found**

Triticum aestivum

>gi|8886324|gb|AAF80449.1|AF161718_1 Sec61p [Triticum aestivum]
MAGGFRVLHLVRPFLAFLPEVQSADRKIPFREKVIYTVISLFIFLVCSQLPLYGIHSTTGADPFYWMRVI
LASNRGTVMELGITPIVTSGMVMQLLVGSKIIEVDNSVREDRALLNGAQKLLGILIAIGEAVAYVLSGMY
GSVSQLGTGNAILIILQLFFAGIIVICLDELLQKGYGLGSGISLFIATNICENIIWKAFSPTTINSGRGA
EFEGAVIALFHLLITRSDKVRALREAFYRQNLPNVTNLLATVLVFLIVIYFQGFRVVLPVRSKNARGQQG
SYPIKLFYTSNMPIILHSALITNLYFISQLLYRKYSGNFLVNLLGIWKESEYSGHSIPVGGLAYYVTAPS
SMADILANPFHALFYVVFMLSACALFSKTWIEVSGSSAKDVAKQLKEQQMVMPGHRESNLQKELNKYIPT
AAAFGGVCIGALTVLADFMGAIGSGTGILLADSIIYQYFETFEKERATELGFFGF

Vitis vinifera

>gi|731439585|ref|XP_010647481.1| PREDICTED: protein transport protein Sec61 subunit alpha-like [Vitis vinifera]
MGGGFRVLHLVRPFLSFLPEVQNADRKIPFREKVIYTVISLFIFLVCSQLPLYGIHSTTGADPFYWMRVI
LASSRGTVMELGITPIVTSGLVMQLLAGSKIIEVDNNVREDRALLNGAQKLLGILIAVGEAVAYVLSGMY
GSVSQLGVGNAILIIVQLCFAGIIVICLDELLQKGYGLGSGISLFIATNICENIIWKAFSPTTINSGRGA
EFEGAVIALFHLLITRTDKVRALREAFYRQNLPNVTNLLATVLIFLIVIYFQGFRVVLPVRSKNARGQQG
SYPIKLFYTSNMPIILQSALVTNLYFISQLLYRRYSGNFLVNLLGKWKESEYSGGQYIPVGGLAYYITAP
SSLADMAANPFHALFYLIFMLAACALFSKTWIEVSGSSARDVAKQLKEQQMVMPGHREANLQKELNRYIP
TAAAFGGMCIGALTVLADFMGAIGSGTGILLAVTIIYQYFETFEKERASELGFFGF

Oryza sativa

>gi|50428644|gb|AAT76995.1| putative Sec61 alpha subunit [Oryza sativa Japonica Group]
MAGRSSSSFRLLDLVRPFMPLLPEVREPDGRRVPFRRKLACTAAALFAFLACSQLPLYGLHRAAAAGGGA
DPFYWVRAILASNRGTVMELGITPVVTAGTLVQLLVGSNLVRADSSNPDDRALLSAAQKLLSIVITAGEA
TAYVLSGAYGSVGVLGAGNAVLVVLQLVLGGMVAIFLDELLQKGYGFGSGISLFTAANTCEGVVTRALSP
ATMDRGRGAEFVGAVTAAAHLLATRARKLSAVREAFFRGGGGGSLPDLRGLAATCAVFLAAVYLQGVRVA
LPVRPRNAPRSHRGGAYSVRLLYTSGMPVVLLSSAVSSLYVVSQALYRRFGGSLLVDLLGKWTPDAAVPV
GGIAYYVTAPASAASAAANPLHAAMYVAFVLAACAALSRAWVVVSGSSSRDVARRLREQQMVMPGMREAS
MQRELERYIPAAAALGGVCVGALTVAADLMGAVGSGAGMLLAVTTVYQCYEAFEKEKTY
